# Angiosperm phylogenetic diversity is lower in Africa than South America

**DOI:** 10.1126/sciadv.adj1022

**Published:** 2023-11-15

**Authors:** Hong Qian, Michael Kessler, Jian Zhang, Yi Jin, Douglas E. Soltis, Shenhua Qian, Yadong Zhou, Pamela S. Soltis

**Affiliations:** ^1^ CAS Key Laboratory for Plant Diversity and Biogeography of East Asia, Kunming Institute of Botany, Chinese Academy of Sciences, Kunming 650201, China.; ^2^ Research and Collections Center, Illinois State Museum, 1011 East Ash Street, Springfield, IL 62703, USA.; ^3^ Department of Systematic and Evolutionary Botany, University of Zurich, Zurich, Switzerland.; ^4^ Center for Global Change and Complex Ecosystems, Zhejiang Tiantong Forest Ecosystem National Observation and Research Station, School of Ecological and Environmental Sciences, East China Normal University, Shanghai 200241, China.; ^5^ Shanghai Institute of Pollution Control and Ecological Security, Shanghai 200092, China.; ^6^ Key Laboratory of National Forestry and Grassland Administration on Biodiversity Conservation in Karst Mountainous Areas of Southwestern China, Guizhou Normal University, Guiyang 550025, China.; ^7^ Florida Museum of Natural History, University of Florida, Gainesville, FL 32611, USA.; ^8^ Genetics Institute, University of Florida, Gainesville, FL 32608, USA.; ^9^ Biodiversity Institute, University of Florida, Gainesville, FL 32611, USA.; ^10^ Department of Biology, University of Florida, Gainesville, FL 32611, USA.; ^11^ Key Laboratory of the Three Gorges Reservoir Region's Eco-Environment, Ministry of Education, Chongqing University, Chongqing 400045, China.; ^12^ College of Environment and Ecology, Chongqing University, Chongqing 400045, China.; ^13^ School of Life Sciences, Nanchang University, Nanchang 330031, Jiangxi, China.

## Abstract

Although originating from a common Gondwanan flora, the diversity and composition of the floras of Africa and South America have greatly diverged since continental breakup of Africa from South America now having much higher plant species richness. However, the phylogenetic diversity of the floras and what this tells us about their evolution remained unexplored. We show that for a given species richness and considering land surface area, topography, and present-day climate, angiosperm phylogenetic diversity in South America is higher than in Africa. This relationship holds regardless of whether all climatically matched areas or only matched areas in tropical climates are considered. Phylogenetic diversity is high relative to species richness in refugial areas in Africa and in northwestern South America, once the gateway for immigration from the north. While species richness is strongly influenced by massive plant radiations in South America, we detect a pervasive influence of historical processes on the phylogenetic diversity of both the South American and African floras.

## Teaser

Differential speciation and extinction in Africa and South America shaped patterns of phylogenetic diversity.

## INTRODUCTION

Africa and South America comprise the two largest tropical continental land masses. Until ~80 to 90 million years (Ma) ago, both were part of the supercontinent Gondwana, and the fossil record shows that their floras were similar at that time (*1*). Until the end of the Cretaceous (66 Ma ago), Africa and South America were both low-lying landmasses, with moist subtropical forests extending from coast to coast (*1*, *2*). At that time, the two continents retained similar floras and were only about 800 km apart, with islands scattered between them, presumably providing opportunities for intercontinental dispersal (*1*). The Cretaceous-Paleogene (K-Pg) mass extinction reshaped floras worldwide (*3*) so that by the Eocene (56 to 34 Ma ago), the floras of Africa and South America had become very different (*1*, *2*). Nearly all of Africa was covered by tropical forest during the early Tertiary, but global cooling and aridification during the Cenozoic resulted in the emergence of the Sahara and other extensive deserts in Africa, and today, tropical forests cover only about 11% of the continent (*4*). In South America, in contrast, the uplift of the Andes restricted the tropical arid regions to a narrow belt along the Pacific coast south of the equator, providing a much larger area for tropical forest (*1*).

Although the contemporary floras of Africa and South America are derived from their common Cretaceous paleoflora of Western Gondwana, today, plant species richness differs substantially between the two continents (*1*, *5*). Africa is 1.7 times as large as South America (30.4 versus 17.8 million km^2^), but the vascular plant species richness of Africa is only about 80% that of South America (65,414 versus 82,052 species) (*6*, *7*). A considerable proportion of African plant diversity is contributed by the highly diverse subtropical Cape flora of southern Africa so that when only the tropical regions of the two continents are compared, the difference in species richness between them is even more evident: tropical Africa (20.0 million km^2^) has 31,323 vascular plant species, whereas tropical South America (13.7 million km^2^) has 79,134 species (*7*). Tropical Africa also has many fewer tree species than tropical South America (*5*, *8*). The disparity in plant species richness between Africa and South America, in general, and their tropical regions, in particular, has long intrigued botanists and biogeographers (*1*, *9*–*11*). Collecting effort may differ between the two continents, but it does not explain the difference in species richness (*10*); South America is likely to have more undescribed tree species than any other continent (*12*).

Several possible factors may explain the differences in plant species richness between Africa and South America (*1*, *10*). Current ecological conditions differ, with Africa, on average, having a more seasonal climate and small areas of very high precipitation (*9*). The geological events that shaped the two continents also differ (*1*). The extent and elevation of the Andes are quite different from mountainous areas in Africa. Tropical mountains, which globally are the main hotspots of plant diversity, are poorly represented in Africa, whereas the Andean chain in South America is the world’s largest tropical mountain system. Thus, Africa may have fewer species because it contains a smaller area of hyperdiverse habitats, namely, perhumid tropical lowland and mountain forests (*13*). Conversely, South America may have higher diversity because it experienced higher diversification rates (the “high speciation hypothesis” of Gentry, 1982), particularly associated with the rise of the Andes, which generated novel habitats and provided opportunity for geographic speciation. In addition, differences in plant diversity between the continents may be the result of historical processes affecting extinction and dispersal. Extinctions, for instance, strongly affected the African flora at the Oligocene-Miocene boundary (23 Ma ago), a period of global aridification (*14*–*16*), as documented by fossil floras (*17*). However, recent simulation analyses suggest that aridification mainly lowered African diversity during the Cretaceous, i.e., shortly after the separation of the two continents, rather than during the Miocene (*11*). Last, South America may also have higher diversity because of the Great American Biotic Interchange (GABI), during which the northern and southern American landmasses came into contact, allowing for the southward migration of Laurasian biotic elements, especially from the boreotropical flora (*18*, *19*). Most likely, all of these factors (i.e., diversification, extinction, and migration) acted in combination (*1*, *10*), but their relative impacts remain poorly known.

Phylogenetic approaches have been applied to investigate and quantify the spatial distribution of lineages across a region of interest. A critical measure used in spatial phylogenetic studies is phylogenetic diversity (PD), which estimates the shared ancestry of species in a region and can be used to identify geographic regions of high lineage diversity after controlling for species richness (*20*). More specifically, the PD of a geographic location is the sum of branch lengths on a phylogenetic tree connecting the terminal taxa present at that particular location (*21*). As pointed out by previous authors [e.g., (*22*, *23*)], on a regional geographic scale, PD often closely corresponds to measures of species richness. However, several factors can lead to significant differences between these measures, including variation in speciation and extinction rates, as well as differences in the biogeographic history of lineages. For example, a rapid and recent diversification may lead to relatively low PD but high species richness. On the other hand, rare long-distance dispersal events between separated regions of different evolutionary histories can substantially increase PD but have a minor effect on species richness. Higher extinction in one region compared to another region can also affect these measures. Using PD metrics to differentiate these processes can be challenging, but analyzing changes in PD over time can be illuminating in this regard: Whereas shifts in diversification rates are simultaneously reflected in a phylogeny and the derived metrics, extinction and immigration can show earlier phylogenetic signals, depending on the placement of the pruned and added phylogenetic branches (fig. S1). In the case of South America and Africa, higher diversification in the former thus will be seen as increasing PD well after the breakup of the Gondwana supercontinent, whereas extinction and immigration can be detected as shifts in PD around the time of the breakup.

Comparisons of the floras of Africa and South America to date have focused largely on species richness (*1*), with phylogenetic studies mostly being limited to specific plant groups [e.g., (*16*, *24*, *25*)]. However, studies of PD of floras at a broad scale can provide important information on the evolutionary and ecological histories of those floras that species richness alone cannot [e.g., (*26*–*28*)]. To date, analyses of PD and the diversification history of the floras of either South America or Africa are few, and comparative analyses between the continents are lacking. De Souza Cortez *et al.* (*29*) examined patterns of temporal diversity and PD in South America, Africa, and Australia using novel metrics to capture plant community age, and Dagallier *et al.* (*30*) examined PD in tropical Africa, but neither study conducted intercontinental comparisons. Hagen *et al.* (*11*) modeled the evolution of biotic diversity across the three main tropical continental land masses, not only largely based on vertebrates but also including some information on plants, concluding that current climate cannot explain their differences in diversity but that high biodiversity in Neotropic and Indomalaya is related to mountain uplift, whereas lower diversity in Africa is associated with lower speciation rates and higher extinction rates linked to Cenozoic aridification. When species richness is controlled statistically, an analysis based on PD can determine whether a difference in species richness between regions is associated with a difference in evolutionary history (*20*). Furthermore, PD may be correlated with the diversity of anatomical, physiological, and behavioral traits so that phylogenetically diverse assemblages tend to be functionally more diverse and correspondingly more stable and resilient (*31*, *32*).

To investigate further the differences between the floras of South America and Africa, we undertook a broad assessment of PD within and between the two continents, both in their entirety and in their climatically matched tropical regions, by comparing the distribution of PD versus species richness for angiosperms. We collated comprehensive plant databases including all known angiosperm species in Africa and South America and identified climatic variation across both continents. The primary objective of this study was to assess to what extent the differences in angiosperm species richness between Africa and South America are also reflected in PD patterns and what these patterns can tell us about the historical causes of floristic differences between the continents.

In the first set of analyses, we asked whether PD of angiosperms differs between the two continents after accounting for species richness. On the basis of the marked differences in species richness and floristic composition between Africa and South America, we predicted that such differences would occur. We then asked whether these differences might be explained exclusively by present-day environmental conditions, in which case, the relationships between PD and environmental conditions should not differ between regions on the two continents where environmental conditions are similar. If differences are found, which we considered to be likely (*11*), then these point to an influence of historical processes.

In the second set of analyses, we analyzed the diversification dynamics of the African and South American floras using PD-through-time (PDTT) plots, which depict the accumulation of PD that has present-day representatives in the respective floras (*33*). We expected that if the differences between the continents are primarily driven by higher diversification rates in South America, then the differentiation in PD between the continents would gradually accumulate over time, particularly within the last 20 to 30 Ma associated with the Andean uplift (fig. S1). Conversely, if the differences were due to survival of Gondwanan lineages that went extinct in Africa or due to immigration of non-African lineages to South America, then PD would show an abrupt shift around the time when the extant lineages in the continents had their last common ancestors, generally around the time of the Gondwanan breakup (fig. S1).

We complemented a phylogenetic approach that quantifies deviations of PD (PD_dev_) relative to the species richness of an area (i.e., normalized PD minus normalized species richness) (*34*–*36*) to identify regions in each continent with phylogenetically unique floras. We predicted that high PD_dev_ would be observed in refugial areas because of the survival of relictual lineages, as well as in areas of possible recent extensive migration [e.g., northwestern South America due to the combination of northern and southern lineages in the area following the Great American Biotic Interchange (*37*, *38*)] (fig. S1). Conversely, we predicted low PD_dev_ in regions where high richness resulted from recent radiation because radiations lead to high species richness while adding little to PD.

## RESULTS

Species richness and PD of angiosperms were strongly correlated (Spearman’s rank correlation = 0.981 for botanical countries in Africa and 0.984 for botanical countries in South America). As a result, geographic patterns of these two metrics of diversity were similar (fig. S2).

PD of angiosperms for South America was 1.4 times that for Africa regardless of whether the two continents as a whole or only their climatically matched tropical regions were compared. There is no area of high PD in Africa (fig. S2B). Of the top 10 families with the highest PD in Africa, five (Asteraceae, Fabaceae, Poaceae, Orchidaceae, and Rubiaceae) are also among the top 10 families with the highest PD in South America (table S1). Species in these five families account for 25.3 and 27.8% of total PD of the angiosperm floras in Africa and South America, respectively. When their climatically matched tropical areas were considered, of the top 10 families with the highest PD in tropical Africa, six (the aforementioned five plus Melastomataceae) are among the top 10 families with the highest PD in tropical South America (table S1). Species in these six families account for very similar values of PD, 30.7 and 28.1% of total PD of the angiosperm floras in tropical Africa and tropical South America, respectively. Of the 211 families of angiosperms that occur in both Africa and South America, Africa has 65,283 species in 5018 genera and South America has 81,253 species in 4108 genera (table S2). Of the remaining families occurring in either continent, 63 and 61 occur in Africa and South America, respectively (table S3); these families have 1208 species in 144 genera and 1795 species in 124 genera, respectively.

**Fig. 1.**
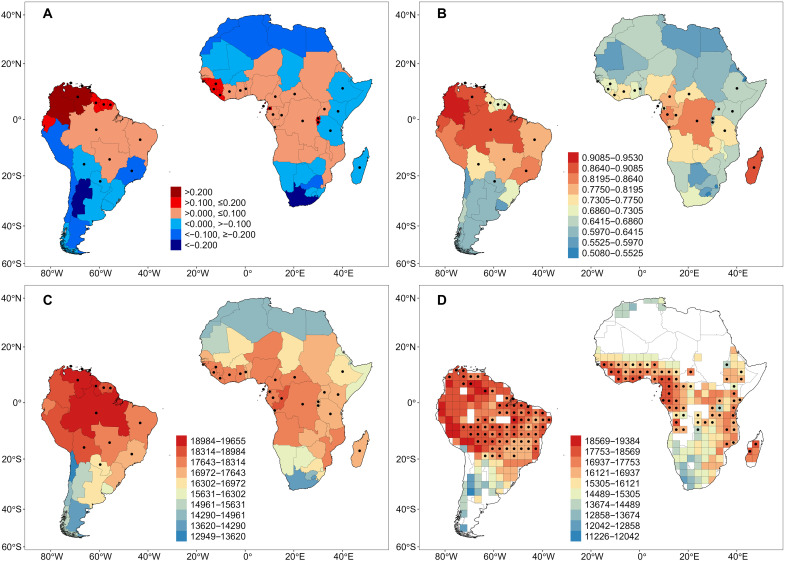
Patterns of PD of angiosperm species in Africa and South America. (**A**) Deviation between area-corrected PD and area-corrected species richness (PD_dev_). (**B**) RPD. (**C**) Standardized PD (i.e., PD per 500 species) in each botanical country. (**D**) Standardized PD (i.e., PD per 500 species) in 300 km–by–300 km grid cells. Botanical countries or grid cells located in climatic conditions matched between tropical Africa and tropical South America were indicated with black dots.

The PD_dev_ analyses revealed areas of higher than expected (based on species richness) PD in northwestern South America (Colombia and Venezuela, less so in Ecuador and the Guianas) and in three areas of Africa: westernmost tropical Africa (Guinea, Sierra Leone, and Liberia), Equatorial Guinea, and Rwanda and Burundi (Fig. 1A). In contrast, we found lower than expected PD in the southern tropical and temperate Andes, as well as in southeastern Brazil, and in the Sahara region, as well as southern Africa.

The results from our analyses of relative PD (RPD; Fig. 1B), which is the ratio of PD observed on the original tree to PD observed on a comparison tree with equal branch lengths, with both trees being scaled such that branch lengths are calculated as a fraction of the total tree length (*28*), complement the results for PD noted above while providing additional insights. There is a broad general trend of high or relatively high PD across the tropics on both continents when standardized PD (i.e., each assemblage including 500 species) is compared, but high values of PD extend well outside the tropical regions (Fig. 1C). In Africa, relatively high standardized PD is observed well north and south of the tropics; in South America, more than half of the continent has high values of PD (Fig. 1C). Our analyses of RPD provide some additional fine-scale perspectives (Fig. 1B). Areas in red-orange and orange (Fig. 1B) have a concentration of significantly longer branches than expected; areas in blue and light blue have a concentration of significantly shorter branches than expected. Those areas of particularly high RPD (Fig. 1B) are more narrowly restricted to what are generally considered areas harboring tropical forests. What is also noteworthy is the much larger extent of regions of high RPD in South America compared to Africa. High RPD in Africa is largely confined to central Africa (e.g., Democratic Republic of the Congo) and especially Madagascar. In South America, high RPD is found across much of the northern and central part of the continent, including Venezuela, Colombia, Ecuador, Peru, and large parts of Brazil. Substantially low RPD occurs in areas of northern and southern Africa and in the southern part of South America (Fig. 1B).

**Fig. 2.**
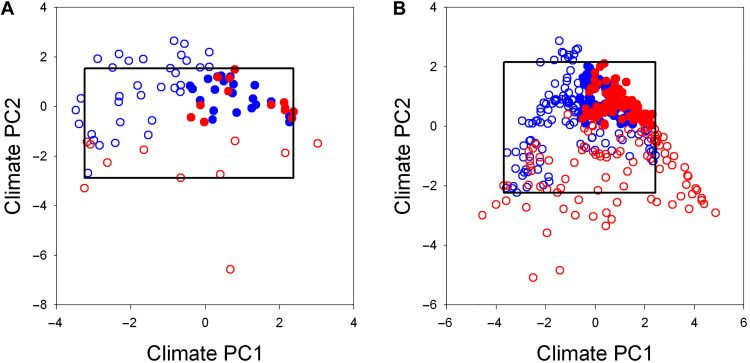
Ordinations of angiosperm assemblages across the first two principal components (PC1 and PC2) of the six climatic variables investigated. (**A**) Angiosperm assemblages in botanical countries. (**B**) Angiosperm assemblages in 300 km–by–300 km grid cells. The inner box in each panel represents the matched climatic conditions between the two continental regions on either axis, and filled circles represent grid cells and botanical countries located in matched tropical climatic conditions (i.e., the upper-left quarter of each inner box).

Of the 78 botanical countries in the two continents (Fig. 1 and fig. S2), 60 (76.9%) were located in areas with climatic conditions that matched between the two continents [i.e., those indicated with dots in Fig. 1 (A to C) and fig. S2 and those located within the inner box of Fig. 2A]. Of the 344 grid cells in Africa and South America that were included here, 290 (84.3%) were in areas with climatic conditions that matched between the two continents (i.e., those indicated with dots in Fig. 1D and located within the inner box of Fig. 2B). Geographic patterns in variation of standardized PD were generally consistent between botanical countries and grid cells within each continent (compare Fig. 1C with Fig. 1D). When standardized PD and standardized mean pairwise distance (MPD; which represents the mean phylogenetic distance between all pairs of species in an assemblage) of angiosperms in the botanical countries and grid cells with matched climatic conditions were compared between the two continents, with variation in current climate and topography accounted for in the analysis of covariance, they both were higher in South America than in Africa in all cases. Furthermore, the differences were significant (*P* < 0.05) in all comparisons, regardless of whether botanical countries or grid cells were considered (Table 1 and tables S4 to S6).

**Table 1.** Analysis of covariance of standardized PD and standardized mean phylogenetic distance (based on 500 angiosperm species randomly selected from each grid cell) in Africa and South America with region (AF versus SA) as the main effect and climate variables (all six PCs) and topographic heterogeneity as covariates. Species assemblages included in this analysis were under matched climate conditions between the two continents (i.e., grid cells located in the inner box of Fig. 2B). Note that data of PD used in the analyses were in the unit of 1000 Ma, and data of mean phylogenetic distance used in the analyses were in the unit of million years. AF, Africa; SA, South America; TOPO, topographic heterogeneity.

	Tropical and extratropical				Tropical only			
Source	SS	df	*F*	*P*	SS	df	*F*	*P*
**Standardized PD (AF < SA)**								
Continent	19.7	1	30.2	<0.001	9.7	1	19.6	<0.001
PC1	191.3	1	293.7	<0.001	8.8	1	17.8	<0.001
PC2	8.5	1	13.1	<0.001	0.8	1	1.6	0.210
PC3	5.5	1	8.4	0.004	0.1	1	0.1	0.726
PC4	0.2	1	0.3	0.581	0.1	1	0.2	0.675
PC5	1.7	1	2.7	0.104	0.9	1	1.8	0.181
PC6	0.9	1	1.4	0.236	0.3	1	0.5	0.468
TOPO	24.0	1	36.8	0	5.8	1	11.6	0.001
Error	183.1	281			67.4	136		
**Standardized mean phylogenetic distance (AF < SA)**								
Continent	35.5	1	5.8	0.016	24.9	1	5.3	0.023
PC1	530.3	1	87.4	<0.001	37.1	1	7.9	0.006
PC2	26.5	1	4.4	0.037	0.6	1	0.1	0.715
PC3	7.0	1	1.2	0.283	19.2	1	4.1	0.045
PC4	9.2	1	1.5	0.220	10.9	1	2.3	0.131
PC5	42.9	1	7.1	0.008	13.9	1	3.0	0.087
PC6	89.6	1	14.8	<0.001	0.4	1	0.1	0.766
TOPO	88.8	1	14.6	<0.001	6.8	1	1.5	0.230
Error	1705.1	281			639.8	136		

Of the 60 botanical countries and 290 grid cells with matched climatic conditions between Africa and South America, 34 and 145, respectively, were characterized as having tropical climatic conditions (21 and 13 botanical countries and 70 and 75 grid cells in tropical Africa and tropical South America, respectively; Fig. 1). Standardized PD in each botanical country or grid cell was, on average, significantly larger under tropical climatic conditions than under extratropical climatic conditions within each continent (17,668 versus 15,993 Ma for botanical countries and 16,786 versus 15,553 for grid cells in Africa; 18,175 versus 16,079 for botanical countries and 17,615 versus 16,800 for grid cells in South America; *t* test, *P* < 0.05 in all cases).

**Fig. 3.**
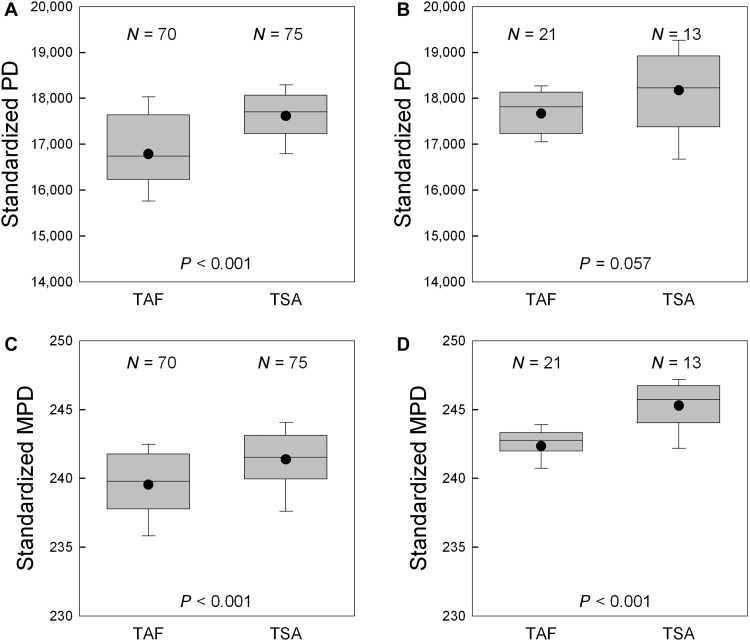
Comparison of standardized PD and standardized MPD of angiosperm species in climatically matched areas between tropical Africa and tropical South America. (**A** and **C**) Standardized PD and standardized MPD of angiosperm species (i.e., PD and MPD per 500 species) in 300 km–by–300 km grid cells. (**B** and **D**) Standardized PD and standardized MPD of angiosperm species (i.e., PD and MPD per 500 species) in botanical countries. *Y* axes are in the unit of million years. The dots represent the mean. Boxes represent the median and 25th and 75th percentiles, and whiskers represent the 10th and 90th percentiles. *P* values were derived from *t* test. TAF, tropical Africa; TSA, tropical South America.

Standardized PD and standardized MPD of angiosperm assemblages with matched tropical climatic conditions between Africa and South America were higher in South America than in Africa in all cases, and the differences were very strongly supported (*P* < 0.001) in three of the four comparisons and moderately supported (*P* = 0.057) in the other comparison (Fig. 3). When standardized PD and standardized MPD of these angiosperm assemblages were compared between the two continents, with variation in current climate and topography accounted for in the analysis of covariance, both standardized PD and standardized MPD were significantly higher in South America than in Africa, regardless of whether botanical countries or grid cells were considered (*P* < 0.05 in all cases; Table 1 and tables S4 to S6). This result derived from the analysis of covariance was consistent with that derived from spatial autoregressive analysis, in which either standardized PD or standardized MPD of the two continents was simultaneously regressed on the climatic and topographical variables. The residuals of each regression for South America were, on average, significantly higher than those for Africa, regardless of whether standardized PD or standardized MPD was considered (for climatically matched grid cells in tropical climate, 270 versus −269 for PD and 0.438 versus −0.507 for MPD; *t* test, *P* < 0.01 in both cases).

**Fig. 4.**
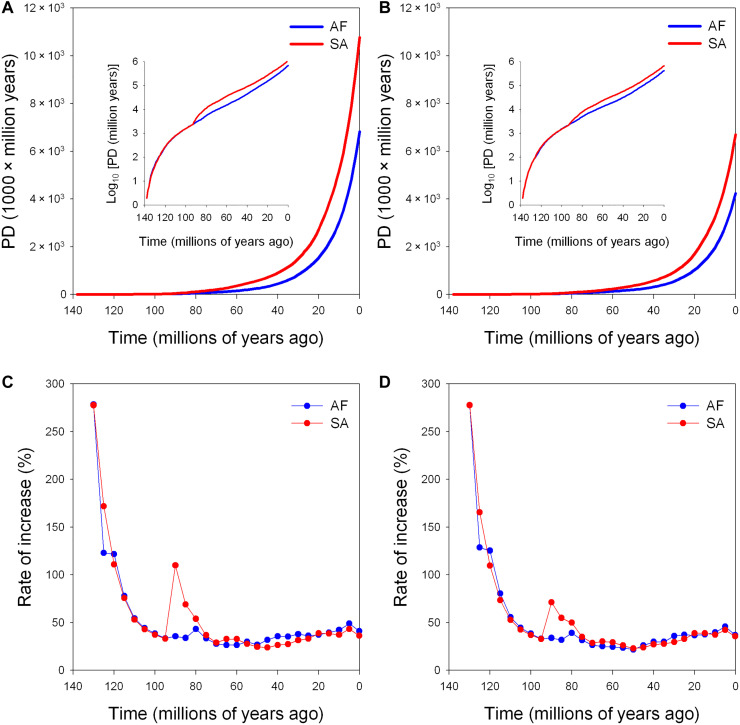
PD-through-time (PDTT) plots and the rate of the increase of PDTT (calculated for each time slot of 5 Ma). (**A** and **C**) All endemic angiosperm species in either Africa or South America. (**B** and **D**) Only those angiosperm species endemic to either Africa or South America that were present in their respective tropical botanical countries indicated in Fig. 2. Insets show the relationships between PD in the logarithm scale and time.

The PDTT plots showed that rates of increase of PD on both continents were very high until about 100 Ma ago but stabilized at similar levels on both continents since then, except for a marked peak in South America ~80 to 90 Ma ago (Fig. 4). Both the total phylogenetic beta diversity and the turnover component of phylogenetic beta diversity (i.e., phylogenetic turnover) between Africa and South America tended to increase linearly across geological time from ~80 Ma ago toward the present (fig. S3).

## DISCUSSION

The differences in floristic composition and species richness of the angiosperm floras of South America and Africa have long been recognized [reviewed in (*1*)], but how these differences are reflected in patterns of PD and what they can tell us about the evolution of these distinct floras have remained unexplored until now. Collectively, our phylogenetic results are generally in agreement with observations on species richness; coupled with the geologic history of the two continents (reviewed above and summarized again below), they provide important insights into the history of the angiosperm floras of South America and Africa. Our study complements Hagen *et al.* (*11*), who used modeling approaches to assess the influence of current and past climate, orogeny, and other factors on the evolution of biotic diversity on the three main tropical land masses. They similarly concluded that low African species diversity was due to low diversification and high extinction linked to aridification, but they did not consider phylogenetic relationships.

We found that PD and RPD show patterns of both similarity and differences. For a given species richness, PD is higher in South America than in Africa. Values of PD in both South America and Africa are highest in tropical areas, in agreement with studies of species richness (*1*). Furthermore, these differences in high PD remain after accounting for current climate. It has been proposed that Africa may have lower plant diversity because of differences in current climate (*10*, *13*). However, we found that the differences in PD are maintained even when accounting for differences in current climate, topographic heterogeneity, and species richness between the continents. Thus, although differences in current climate and other factors may certainly play a role in shaping the floras of the two continents, our study also reveals a strong signal of historical processes. Because of the paucity of data on many historical processes in Africa and South America, we did not explore how past environments and past geological events have affected the current status of PD of angiosperms. This approach was taken by Hagen *et al.* (*11*), but while their results are partly congruent with our findings, the paleoclimatic models that they used are highly simplified. Future studies may better address this question when more data become available.

Areas of very high RPD (Fig. 1) are particularly informative. These RPD patterns, which indicate a concentration of long phylogenetic branches, make sense biologically. RPD is highest in areas of the tropics; these are areas harboring tropical forests: areas that are rich in highly diverse lineages that broadly span the angiosperm branch of the tree of life. Hence, these areas have much higher RPD than surrounding areas. What is also noteworthy is the much larger extent of regions of high RPD in South America compared to Africa (Fig. 1), suggesting that there are more species in the South American tropics because of the more extensive tropical forest areas that harbor particularly large cross sections of the angiosperm tree of life.

Our results are also informative when considered in the context of other recent work that helps place our PD metrics into a broader perspective. Some of the tropical areas identified here as having high PD or RPD correspond to areas inferred to be of old community age (*29*), specifically those communities in tropical areas bordering the Andes and in some areas of tropical Africa, reinforcing the suggestion supported here that these areas harbor many diverse lineages. These findings for Africa also agree with the PD analyses of Dagallier *et al.* (*30*), who found higher RPD in the Guineo-Congolian region of Africa, supporting the hypothesis that African lowland forests are home to some of the oldest, most phylogenetically diverse lineages in Africa (*16*, *29*).

Areas of low PD and RPD also can be placed in a broader historical context. Areas of low RPD are found in northern Africa and the southern tip of southern Africa, as well as the very southern part of South America and along parts of the Andes (Fig. 1). These spatial phylogenetic findings are consistent with areas known to have supported recent radiations yielding closely related species [e.g., (*16*, *39*, *40*)].

The timing of various factors influencing the floras of South America and Africa was estimated using PDTT plots, which are analogous to lineage-through-time (LTT) plots (*41*), but instead of accumulating the number of phylogenetic lineages over time, they revealed the accumulation of PD over time. The interpretation of LTT and PDTT plots is challenging and should be made with caution because they are based only on extant lineages, so that extinct lineages are excluded, and because a given LTT pattern can emerge from different historical processes (*42*, *43*). Nevertheless, they allow discrimination of signals of higher diversification versus extinction or immigration (fig. S1).

The results of our PDTT analyses were not consistent with a model in which the current differences in PD between Africa and South America are primarily the result of increased diversification in South America (*44*). Rather, we found that the differences in PD between the continents are primarily reflected as abrupt increases in both the rate of increase of PD and the absolute PD in South America around the timing of the Gondwanan breakup 80 to 90 Ma ago, with similar rates of increase of PD on both continents at earlier and later times. This pattern is likely the result of extinction in Africa and/or of immigration to South America. Extinction played a major role in shaping the African flora, particularly in association with global aridification especially during the Miocene (*14*–*16*) with tropical forests only surviving in refugial areas. For instance, the late Oligocene fossil leaf flora of Chilga (Ethiopia) has a diversity comparable to that of modern South American leaf assemblages, whereas an early Miocene flora from the same region is massively impoverished (*17*). Beyond the shift that occurred 80 to 90 Ma ago, some of the increased PD in South America may be due to more recent processes. Immigration into South America from North America via the GABI occurred within the past 5 to 10 Ma or earlier because of island-hopping (*45*), when the developing land bridge between South America and Mesoamerica allowed for the immigration of diverse faunal and floristic elements, including many associated with the boreotropical and north-temperate floras, such as the tree genera *Alnus* (Betulaceae) and *Magnolia* (Magnoliaceae) (*37*, *46*). No similar intercontinental floristic interchange occurred in Africa.

That these extinction and immigration events are not recovered in the PDTT plots at the actual time of these events may have to do with the structure of the phylogenetic trees. For instance, if a lineage became extinct in Africa 30 Ma ago while surviving in South America, its “appearance” in South America would be dated at the point of the last common ancestor, most likely in the common Gondwanan flora (fig. S1). A similar reasoning applies to the phylogenetic signal of immigration to South America, where the phylogenetic connection of a new lineage may also be much earlier than the actual immigration event but with an important difference. For instance, oaks (*Quercus*, Fagaceae) colonized South America from Mesoamerica ~2 Ma ago and have spread through the Colombian Andes (*47*, *48*), while the family, which is otherwise not represented in South America, is around 90 Ma old (*49*), so that the placement of the oak species in the phylogeny would indicate an appearance at around this time. Hagen *et al.* (*11*) found that low vertebrate diversity in Africa was more strongly related to aridity in the Cenozoic right after the continental breakup than by the more recent Miocene aridification. However, fossil floras and presumably the climate were similar on the two continents (which were only 800 km apart) until the end of the Cretaceous (66 Ma ago) (*1*, *2*). It is thus possible that the Cenozoic signal found by Hagen *et al.* (*11*) is due to the effect that we described above, namely, that recent extinctions are recovered in phylogenies at earlier times.

The influence of extinction in Africa and of immigration into South America from the north is also revealed by the PD_dev_ analysis. In Africa, we found three hotspots of high PD relative to the number of species known from these countries. All three hotspots correspond to well-known refugial areas with high endemism (Guinea Coast, Bight of Biafra, and Ruwenzori/Virunga mountains), where tropical forests persisted even in arid geological periods such as the mid-Miocene (*50*). The latter two refugial areas were recovered by us only in small countries (Equatorial Guinea, Rwanda, and Burundi) that do not fully cover the distribution of the refugial areas. One might thus assume that this pattern is an artifact of country size, although our analytical approach accounted for area. We consider this unlikely because other small countries such as Togo, Eritrea, Lesotho, or Swaziland do not show this effect. Rather, we suggest that in large countries where refugial areas only cover a small part of the country, their phylogenetic signature is averaged out by the spatially dominant flora. Thus, we predict that other well-known refugial areas such as the mountains in Cameroon or the Tanzanian portion of the Eastern Arc mountains (which range from Kenya to Malawi) would also have high positive values of PD_dev_, if analyses were conducted at a finer spatial scale.

In contrast to these hotspots of PD_dev_, we found notably low values in southern Africa, in agreement with our findings for RPD. The Cape flora is well known for its extremely high species richness and taxonomic distinctness so that it is recognized as a distinct floristic kingdom (*40*). However, this is the result of radiations within the past 20 Ma of relatively few plant lineages such as the families Iridaceae, Proteaceae, and Restionaceae or the genera *Erica* and *Phylica* (*51*). Recent radiations led to an accumulation of species with little added PD.

In South America, we found notably high PD_dev_ in the northwest, where plant lineages colonizing the continent from the north would have arrived first via the Great American Biotic Interchange, followed by limited dispersal into the rest of South America. In contrast, we found negative values of PD_dev_ in the southern tropical Andes (Peru and Bolivia) and in southeastern Brazil, although these areas harbor some of the world’s richest floras. In parallel with Africa’s Cape region, we propose that limited PD in these regions is the result of regional radiations that increased species richness while adding little PD. Such regional radiations have been documented in southeastern Brazil in the families Asteraceae (*52*), Bromeliaceae (*53*), and Gesneriaceae (*54*), as well as many others.

In conclusion, we show that patterns of species richness and PD in South America and Africa show some important similarities on the one hand and informative differences on the other hand. Our study showed that PD is higher in tropical South America than in tropical Africa with similar climatic conditions after accounting for species richness and topographic heterogeneity, as previously shown for species richness (*1*, *11*). However, whereas species richness is strongly influenced by massive recent radiations of numerous plant lineages in South America [e.g., (*16*, *39*, *40*)], these radiations only add many short phylogenetic branches, which do not greatly add to PD. PD, in turn, shows strong effects of historical processes, namely, extinction of Gondwanan plant lineages in Africa following the continental breakup, and immigration to South America from North America of plant lineages during the GABI. These processes resulted in lower PD in Africa overall but with local hotspots in refugial areas where tropical forests and phylogenetically distinct plant lineages survived arid periods.

## MATERIALS AND METHODS

### Species distribution data

We used two sets of species lists of angiosperm assemblages. One set of species lists was obtained from “botanical countries” defined by the World Geographical Scheme for Recording Plant Distributions (*55*), as shown in fig. S2 for the botanical countries in Africa and South America. Species lists for the botanical countries are commonly used in ecological and biogeographic studies [e.g., (*56*, *57*)]. There are 54 and 24 botanical countries located in Africa and South America, respectively (fig. S2). Angiosperm native species lists for the botanical countries were derived from World Plants (WP; www.worldplants.de) and World Checklist of Vascular Plants (WCVP; available at www.plantsoftheworldonline.org) (*58*), using data download scripts [e.g., rWCVP; (*59*)]. Infraspecific taxa were combined with their respective species. As a result, 147,439 angiosperm species were included in our combined species list for Africa and South America.

The other set of species lists was derived from species occurrence data. We extracted species occurrences for angiosperms in Africa from the Global Biodiversity Information Facility (GBIF; www.gbif.org) and tropical African vascular plants (RAINBIO; https://gdauby.github.io/rainbio/index.html) and species occurrences for angiosperms in South America from GBIF and the Botanical Information and Ecology Network (BIEN; http://bien.nceas.ucsb.edu/bien/). If an occurrence represents a species occurring in a country but the species was not treated as native to the country according to WP and WCVP, then we excluded the occurrence. We standardized botanical nomenclature according to WP using U.Taxonstand (*60*). Infraspecific taxa were combined with their respective species. We generated species lists from the occurrence data for equal-area regions (grid cells). Species lists derived from occurrence data with these data sources are generally incomplete, but the incompleteness decreases with increasing spatial scale (*6*). Qian *et al.* (*6*) showed that a species list derived from GBIF and RAINBIO for a grid cell of 300 km by 300 km includes the majority (72% on average) of the species in the grid cell in Africa. Accordingly, we used the occurrence data to generate species lists at the scale of 300 km by 300 km for Africa and South America using the Albers equal-area projection. Because our study was based on a fixed number of species randomly drawn from a species assemblage (see below for details) and because each species assemblage included, on average, most of the species in the assemblage, the occurrence-based species lists are appropriate for our study. Grid cells that include less than 50% of their area on land were excluded from this study.

### Phylogeny reconstruction and PD

We used the V.PhyloMaker2 package [build.nodes.1 (*61*); also see (*62*)], which is based on functions reported by Jin and Qian (*63*), to generate a phylogeny for the species in our dataset using GBOTB.extended.WP.tre, which is an updated and expanded version of the dated megaphylogeny GBOTB reported by Smith and Brown (*64*) [see (*63*) for details], as a backbone. We added the genera and species in our dataset that were absent from the megaphylogeny to their respective families and genera using V.PhyloMaker2 software (scenario 3) (*61*). Specifically, V.PhyloMaker2 sets branch lengths of added taxa in a family by placing the nodes evenly between dated nodes and terminals within the family and placing a missing species at the midpoint of the branch length of its genus (*63*). We pruned the megaphylogeny to generate a phylogenetic tree retaining only the angiosperm species present in our dataset. Using such an approach to generate plant phylogenetic trees is common in recent ecological and biogeographic studies [e.g., (*65*–*68*)], and a recent study (*69*) has shown that phylogenetic structure metrics estimated on a tree resolved to genera are almost indistinguishable from those estimated on a tree resolved at the species level. We found that the distributions of unresolved species across age classes are very similar between Africa and South America (Spearman’s rank correlation = 0.940; fig. S4), suggesting that the phylogeny used in this study has no systematic bias toward either continent in terms of determining which continent has a higher PD.

We used Faith’s (*21*) PD as a metric of PD in each angiosperm assemblage. PD, which represents the sum of the branch lengths of the phylogenetic tree linking all species of a particular assemblage, consistently increases with species richness in an assemblage (*23*). To account for this effect of species richness, we took a rarefaction approach to calculate a standardized PD measure (*20*). Specifically, for each angiosperm flora (i.e., a botanical country or grid cell), we calculated PD for a set of 500 species randomly selected from the flora, repeating this simulation 1000 times to estimate the mean of randomized PD values. The approach that we used to standardize PD based on a fixed number of species is commonly used in the current literature [e.g., (*20*, *70*–*73*)]. We chose 500 species for each randomized assemblage to maximize the number of species per assemblage on the one hand and to maximize the number of species assemblages on the other hand. Qian *et al.* (*20*) showed that patterns of standardized PD in regional angiosperm floras are nearly identical among four different numbers (200, 400, 600, and 800) of selected species in each assemblage, suggesting that including 500 species in each randomized assemblage is appropriate for regional angiosperm floras. We also calculated MPD as a secondary measure of PD. MPD represents the mean phylogenetic distance between all pairs of species in an assemblage, the phylogenetic distance between a pair of species being defined as the total branch length of the shortest path between the two species. For each angiosperm flora, we calculated MPD for a randomly selected set of 500 species, repeating this simulation 1000 times to estimate the mean of randomized MPD values. We used the software PhyloMeasures to calculate PD and MPD (*74*).

In addition to PD and MPD, we also calculated RPD and the deviation between PD and species richness (PD_dev_). RPD is the ratio of PD observed on the original tree to PD observed on a comparison tree with both trees being scaled such that branch lengths are calculated as a fraction of the total tree length (*28*). The comparison tree retains the topology of the original tree but has all branches in equal length. A high value of RPD indicates an overrepresentation of long branches, whereas a low value of RPD indicates an overrepresentation of short branches. PD_dev_ is defined as normalized PD minus normalized species richness [i.e., mean = 0 and SD = 1 for both PD and species richness after normalization (*34*–*36*)]. This approach is akin to using the relationship between species richness and PD as a null model to account for sampling effects of different levels of species richness among regions when investigating PD (*35*, *75*).

We determined PD at a particular geological time (at an interval of 1 Ma) for species in each of the two continents (and their tropical areas) and generated PDTT plots. We also determined phylogenetic beta diversity between Africa and South America at a particular geological time (also at an interval of 1 Ma) and generated a phylogenetic-beta-diversity-through-time plot. We did so both for the total phylogenetic beta diversity and for the turnover component of phylogenetic beta diversity (i.e., phylogenetic turnover) (*76*). The total phylogenetic beta diversity was measured as the Sørensen dissimilarity index, using the formula (*b* + *c*)/(2*a* + *b* + *c*), and the turnover component of phylogenetic beta diversity was measured as the Simpson dissimilarity index, using the formula min(*b*,*c*)/[*a* + min(*b*,*c*)], where *a* is the shared branch length by the two continents, *b* is the branch length unique to one continent, and *c* is the branch length unique to the other continent (*76*, *77*).

### Climate and topography data

Mean annual temperature, annual precipitation, minimum temperature of the coldest month, precipitation during the driest month, temperature seasonality, and precipitation seasonality represent the mean, extreme, and variability of temperature and precipitation. These six climatic variables have been commonly considered as the most important climatic factors determining distributions and diversity of plants (*27*, *78*–*80*). Accordingly, we used these six climatic variables to characterize the climate of each grid cell or botanical country. We obtained climate data from CHELSA (https://chelsa-climate.org/bioclim; variables bio1, bio12, bio6, bio14, bio4, and bio15) (*81*). The mean value of each of the six variables was calculated for each grid cell or botanical country using data at 30–arc sec resolution.

We subjected the six climate variables for the grid cells and botanical countries in Africa and South America to a principal components analysis (PCA) based on their correlation matrix using PC-ORD version 4 (*82*). The first two climatic axes (PC1 and PC2) accounted for the vast majority (84.2%) of the variance in the six climate variables (table S7); thus, we used PC1 and PC2 to determine climatically matched areas between Africa and South America.

We examined the effect of topographical variation on PD. Elevation range within a sampling area has been commonly used as a measure of topographical variation within the area [e.g., (*83*)]. Elevation range was log_10_-transformed (*84*). We obtained elevation range for each grid cell and botanical country from GMTED2010 (www.usgs.gov/coastal-changes-and-impacts/gmted2010).

### Statistical analysis

We plotted the botanical countries from Africa and South America on climate PC1 and PC2 and determined those botanical countries with matching climatic conditions between the two continents on either PC axis (Fig. 2A). We divided the rectangle with the botanical countries under matching climatic conditions (as shown in Fig. 2A) into four parts and considered those botanical countries located in the quarter of the rectangle with high values of each PC (i.e., higher temperature and precipitation) as representing tropical climatic conditions that are matched between Africa and South America (filled dots in Fig. 1). Using the same approach, we determined those grid cells in which climatic conditions in general and tropical climate conditions in particular match between the two continents (Fig. 2B).

We used analysis of covariance (ANCOVA) to explore differences in PD (i.e., standardized PD and standardized MPD) between Africa and South America, with continent as a main effect and climatic variables and log_10_-transformed elevation range as covariates. ANCOVA was used for climatically matched botanical countries and grid cells located in both tropical and extratropical climates and for those only located in tropical climate. In each case, we did two sets of ANCOVA. In one set, the climatic variables included in the analyses were the scores of the six PC axes derived from the six climatic variables used in this study; this set of analyses precluded or minimized the issue of collinearity among raw climatic variables. In the other set of analyses, we used the raw data of the six climatic variables in each ANCOVA; this set of analyses allowed assessment of the contribution of each raw climatic variable to each ANCOVA, although determining the relative importance of each covariate was not a goal of this study. The ANCOVA approach that we used has been frequently used in previous studies determining differences in diversity between continents [e.g., (*20*, *85*, *86*)].

In addition to using the aforementioned ANCOVA approach to compare standardized PD between Africa and South America, we also used a regression approach to determine and test differences in PD between the two continents. Specifically, we used a spatial autoregressive error model to simultaneously regress standardized PD or standardized MPD derived from angiosperm assemblages in grid cells located in climatically matched tropical climate between the two continents on scores of the six axes of the PCA derived from the six climatic variables and log_10_-transformed elevation range; we used a *t* test to determine whether residuals from the regression differed between the two continents. This method has also been used in previous studies determining differences in diversity between continents [e.g., (*20*, *86*)]. We also used *t* test to compare standardized PD and MPD between tropical Africa and tropical South America. We used SYSTAT version 7 (*87*) and the package “Spatial Analysis in Macroecology” (www.ecoevol.ufg.br/sam/) to conduct statistical analyses.

## Supplementary Materials

### This PDF file includes:

Figs. S1 to S4 Tables S1 to S7 References
