# Supplementary material for: Angiosperm phylogenetic diversity is lower in Africa than South America
Source: Sci Adv. 2023 Nov 15;9(46):eadj1022. doi: 10.1126/sciadv.adj1022 (PMC10651126; doi:10.1126/sciadv.adj1022)
Supplement: Supplementary file 1 — Figs. S1 to S4 Tables S1 to S7 References [file sciadv.adj1022_sm.pdf]

Supplementary Materials for

Angiosperm phylogenetic diversity is lower in Africa than South America

Hong Qian *et al*.

Corresponding author: Hong Qian, hqian@museum.state.il.us; Shenhua Qian, qian@cqu.edu.cn

Sci. Adv. **9**, eadj1022 (2023)
DOI: 10.1126/sciadv.adj1022

This PDF file includes:

Figs. S1 to S4
Tables S1 to S7
References

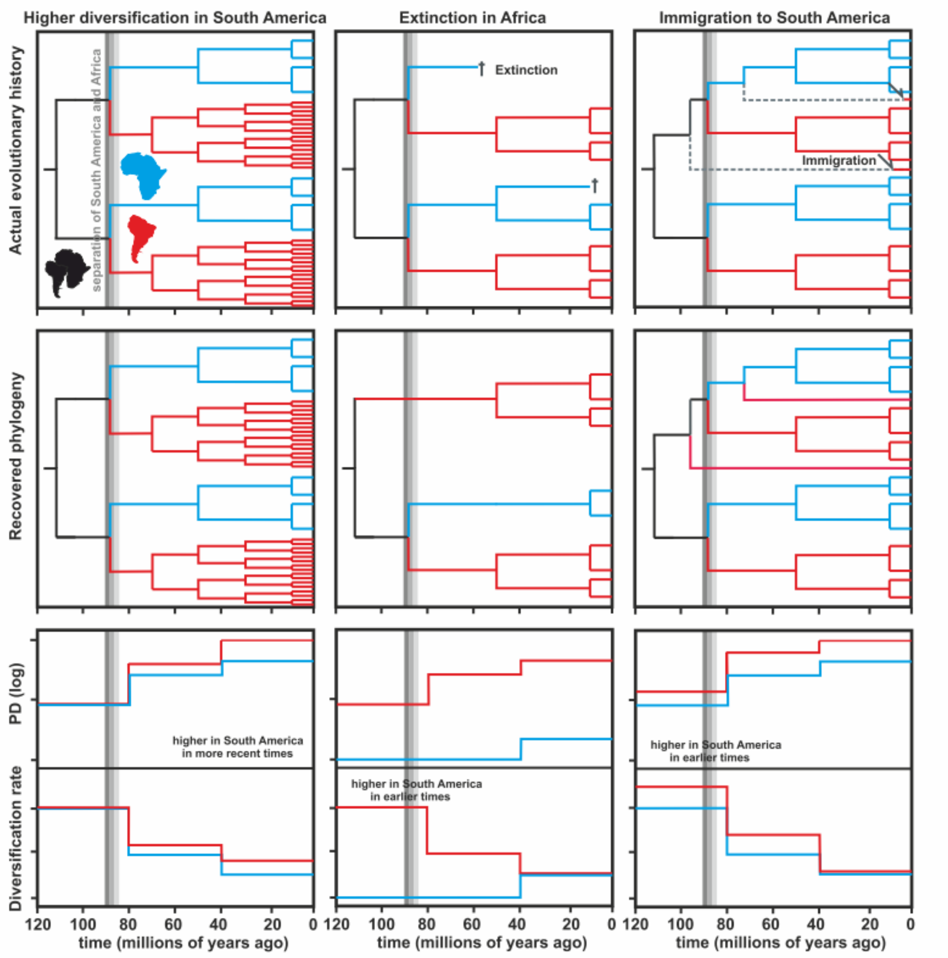

Fig. S1. Three examples showing how different evolutionary processes are reflected in phylogenetic trees and the derived metrics of phylogenetic diversity and rates of increase of phylogenetic diversity. Phylogenetic expectations of different scenarios (diversification, extinction, recent immigration, left to right) that potentially explain differences in PD between Africa and South America. The top row shows the actual evolutionary history, the middle row shows the recovered phylogenetic tree, and the bottom row shows the relationship between diversification rate and phylogenetic diversity (PD). Blue branches = species in Africa; red branches = species in South America. The bold vertical line indicates the timing at which South America separated from Africa. Note that depending on the frequency and timing of extinction and immigration different patterns can emerge, but one pattern remains constant: In the case of increased diversification the resulting signals on phylogenetic diversity and rates must occur simultaneously, whereas in the case of extinction and immigration the signals can occur much earlier, depending on the length of the affected evolutionary branches. Based on our analyses all three factors (diversification, extinction, recent immigration) likely contributed to the differences observed in PD as well as RPD (see text).

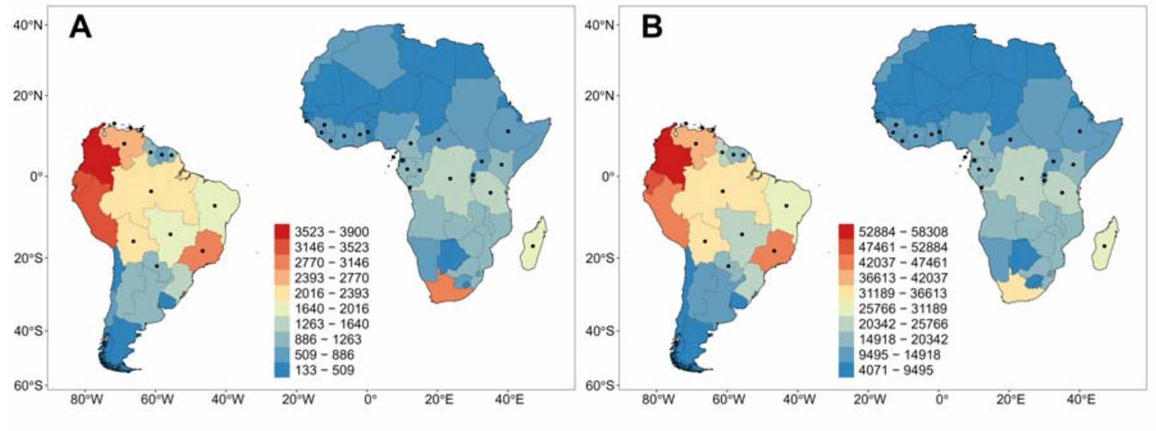

Fig. S2. Area-corrected species richness (A) and phylogenetic diversity (B) of angiosperms in each botanical country in Africa and South America. Area-corrected species richness (or phylogenetic diversity) resulted from species richness (or phylogenetic diversity) being divided by $\log_{10}$-transformed area in square kilometer. Botanical countries located in climatic conditions matched between tropical Africa and tropical South America were indicated with black dots.

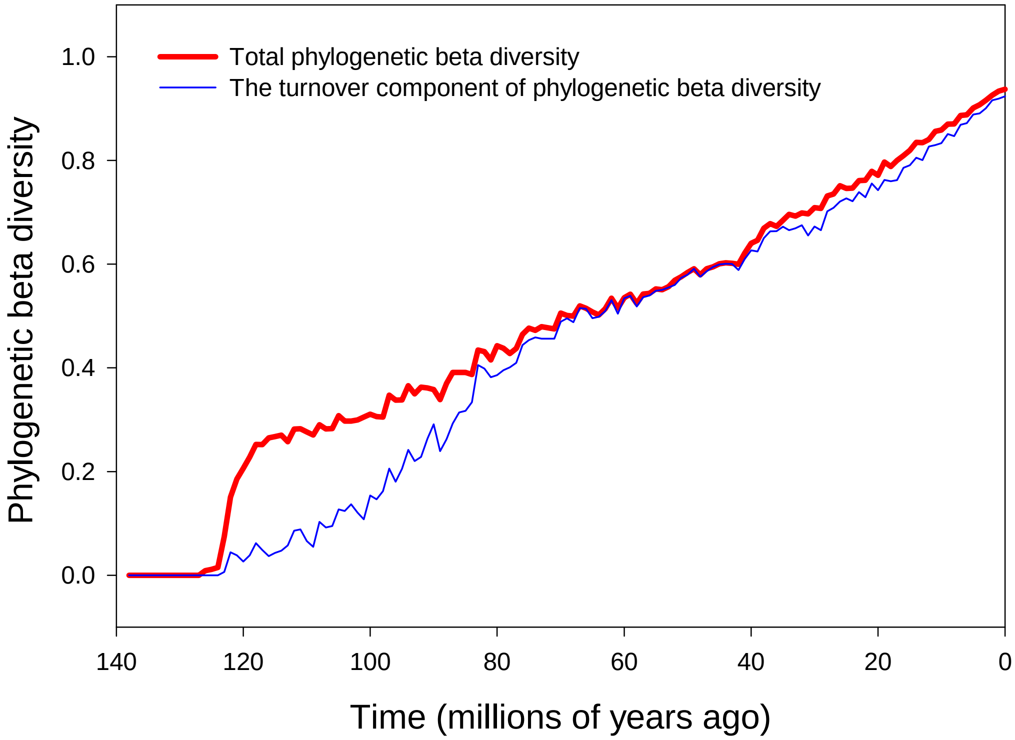

Fig. S3. Phylogenetic beta diversity through time for angiosperms between Africa and South America. Total phylogenetic beta diversity was measured as the Sørensen dissimilarity index, using the formula $(b + c)/(2a + b + c)$, and the turnover component of phylogenetic beta diversity was measured as Simpson dissimilarity index, using the formula $\min(b,c)/[a + \min(b,c)]$, where a is the shared branch length by the two continents, b is the branch length unique to one continent and c is the branch length unique to the other continent. Phylogenetic beta diversity was calculated for each one-million-years time slice across the phylogenetic tree used in the study.

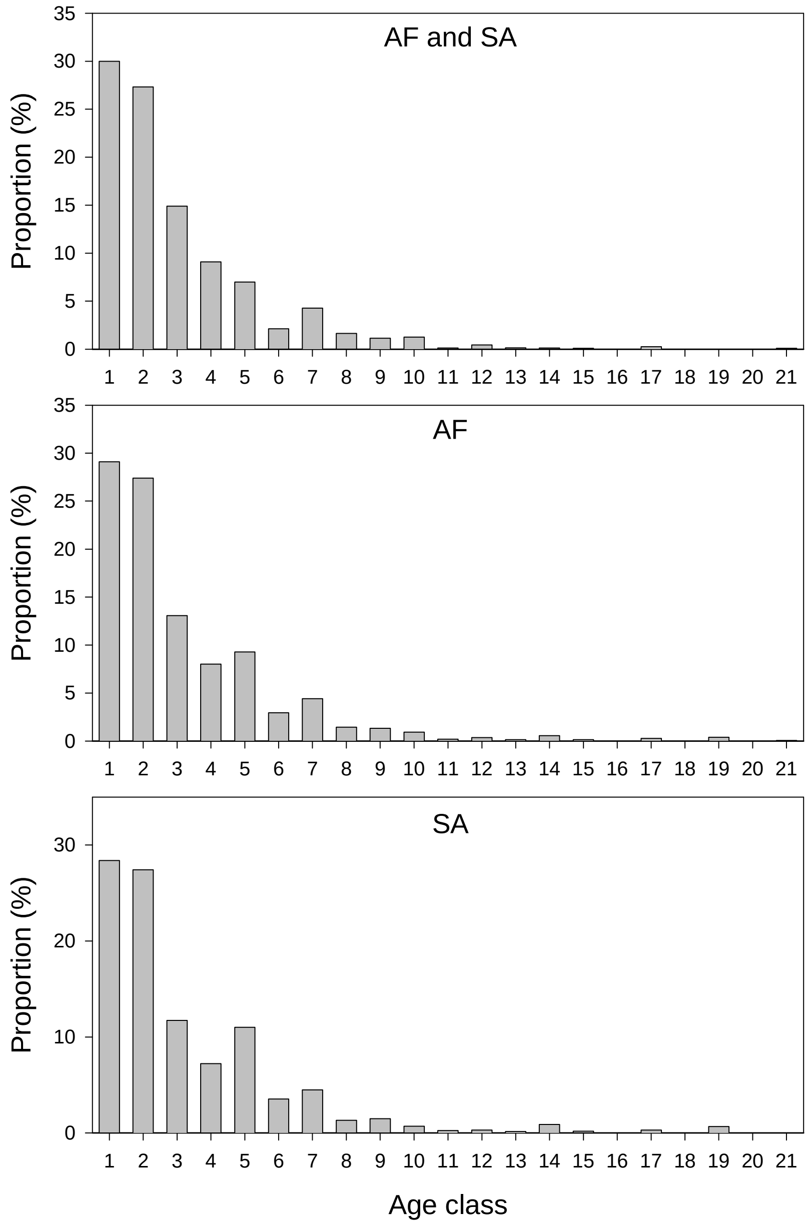

Fig. S4. Proportion (%) of unresolved species in different ages in the phylogenetic tree used in this study on angiosperms in Africa (AF) and South America (SA). Each age class represents five million years except for class 21, which included all species older than 100 million years. The distribution of unresolved species across age classes in Africa is very similar to that of South America (Spearman's rank correlation = 0.940).

Table S1. The top 10 families that have the highest phylogenetic diversity (PD, × 1000 million years) in Africa and South America and in their climatically matched tropical regions. Families and PD were sorted in the descending order of PD.

Africa				South America			
Continent		Tropic		Continent		Tropic	
Family	PD	Family	PD	Family	PD	Family	PD
Fabaceae	75.5	Fabaceae	52.8	Orchidaceae	135.3	Fabaceae	59.4
Orchidaceae	40.9	Orchidaceae	33.9	Araceae	93.1	Orchidaceae	55.4
Asteraceae	31.5	Poaceae	17.9	Fabaceae	75.3	Araceae	41.9
Poaceae	27.9	Acanthaceae	16.7	Piperaceae	51.7	Myrtaceae	29.4
Euphorbiaceae	24.8	Euphorbiaceae	16.7	Asteraceae	41.4	Asteraceae	20.7
Asparagaceae	21.9	Rubiaceae	16.3	Myrtaceae	34.2	Piperaceae	20.3
Acanthaceae	21.7	Asteraceae	14.1	Melastomataceae	27.1	Poaceae	19.3
Rubiaceae	18.5	Malvaceae	11.5	Poaceae	26.6	Melastomataceae	18.1
Malvaceae	16.6	Melastomataceae	9.9	Bromeliaceae	26.4	Rubiaceae	16.9
Aizoaceae	15.8	Lamiaceae	9.9	Rubiaceae	22.4	Bromeliaceae	16.7

Table S2. The numbers of genera and species in the families of angiosperms which occur in both Africa and South America.

	Tropical and extra-tropical		Tropical only	
Continent	Genus	Species	Genus	Species
Africa	5018	65,283	3429	36,388
South America	4108	81,253	3478	49,887

Table S3. Families present in Africa (AF) and absent from South America (SA) or vice versa.

Families present in AF and absent from SA		Families present in SA and absent from AF	
Ancistrocladaceae	Liliaceae	Achatocarpaceae	Marcgraviaceae
Aphloiaceae	Limeaceae	Actinidiaceae	Melanthiaceae
Aponogetonaceae	Lophiocarpaceae	Aextoxicaceae	Microteaceae
Asteropeiaceae	Montiniaceae	Alstroemeriaceae	Misodendraceae
Balsaminaceae	Moringaceae	Alzateaceae	Mitrastemonaceae
Barbeuiaceae	Musaceae	Asteliaceae	Muntingiaceae
Barbeyaceae	Myrothamnaceae	Atherospermataceae	Nartheciaceae
Bruniaceae	Nepenthaceae	Bataceae	Nelumbonaceae
Butomaceae	Neuradaceae	Berberidopsidaceae	Nothofagaceae
Centroplacaceae	Nitrariaceae	Bonnetiaceae	Peltantheraceae
Colchicaceae	Paeoniaceae	Brunelliaceae	Philesiaceae
Ctenolophonaceae	Pandaceae	Calceolariaceae	Phyllonomaceae
Curtisiaceae	Pandanaceae	Calyceraceae	Picramniaceae
Cynomoriaceae	Penaeaceae	Caryocaraceae	Polemoniaceae
Didiereaceae	Pentadiplandraceae	Clethraceae	Quillajaceae
Dioncophyllaceae	Physenaceae	Columelliaceae	Rhabdodendraceae
Dirachmaceae	Pittosporaceae	Corsiaceae	Sabiaceae
Drosophyllaceae	Posidoniaceae	Cyclanthaceae	Sarraceniaceae
Elaeagnaceae	Resedaceae	Cyrillaceae	Schlegeliaceae
Flagellariaceae	Roridulaceae	Dipentodontaceae	Schoepfiaceae
Geissolomataceae	Salvadoraceae	Escalloniaceae	Staphyleaceae
Gerrardinaceae	Sarcolaenaceae	Euphroniaceae	Stylidiaceae
Gisekiaceae	Sladeniaceae	Gomortegaceae	Styracaceae
Grubbiaceae	Sphaerosepalaceae	Goupiaceae	Tapisciaceae
Huaceae	Sphenocleaceae	Griseliniaceae	Tetrachondraceae
Hydrostachyaceae	Stilbaceae	Halophytaceae	Tetrameristaceae
Irvingiaceae	Tamaricaceae	Heliconiaceae	Tofieldiaceae
Iteaceae	Thomandersiaceae	Hydrangeaceae	Tovariaceae
Ixioliriaceae	Tiganophytaceae	Koeberliniaceae	Tropaeolaceae
Kewaceae	Torricelliaceae	Krameriaceae	
Kirkiaceae	Vahliaceae	Lacistemataceae	
Lanariaceae		Lardizabalaceae	

Table S4. Analysis of covariance of standardized phylogenetic diversity and standardized mean phylogenetic distance (based on 500 angiosperm species randomly selected from each botanical country) in Africa (AF) and South America (SA) with continent (AF versus SA) as the main effect and climate variables (all six PCs) and topographic heterogeneity (TOPO) as covariates. Species assemblages included in this analysis were under matched climate conditions between the two continents (i.e. botanical countries located in the inner box of Fig. 2A).

	Tropical and extra-tropical				Tropical only			
Source	SS	df	*F*	*P*	SS	df	*F*	*P*
Standardized phylogenetic diversity (AF < SA)								
Continent	2.1	1	6.6	0.013	1.7	1	8.3	0.008
PC1	50.6	1	155.1	<0.001	6.1	1	29.3	<0.001
PC2	6.9	1	21.2	<0.001	1.1	1	5.2	0.032
PC3	<0.1	1	0.1	0.787	<0.1	1	0.2	0.648
PC4	0.3	1	1.0	0.329	<0.1	1	<0.1	0.929
PC5	0.5	1	1.6	0.208	0.1	1	0.3	0.61
PC6	6.8	1	20.7	<0.001	1.5	1	7.3	0.012
TOPO	1.4	1	4.3	0.044	0.9	1	4.4	0.047
Error	16.7	51			5.2	25		
Standardized mean phylogenetic distance (AF < SA)								
Continent	30.7	1	11.0	0.002	42.0	1	19.7	<0.001
PC1	175.1	1	62.5	<0.001	33.7	1	15.8	0.001
PC2	12.2	1	4.4	0.042	2.7	1	1.3	0.271
PC3	5.3	1	1.9	0.176	1.6	1	0.7	0.402
PC4	0.3	1	0.1	0.735	0.5	1	0.2	0.626
PC5	3.3	1	1.2	0.286	0.2	1	0.1	0.773
PC6	35.4	1	12.6	0.001	1.6	1	0.7	0.397
TOPO	0.8	1	0.3	0.594	2.5	1	1.2	0.287
Error	142.8	51			53.4	25		

Note that data of phylogenetic diversity used in the analyses were in the unit of 1000 million years, and data of mean phylogenetic distance used in the analyses were in the unit of million years.

Table S5. Analysis of covariance of standardized phylogenetic diversity and standardized mean phylogenetic distance (based on 500 angiosperm species randomly selected from each grid cell) in Africa (AF) and South America (SA) with continent (AF versus SA) as the main effect and climate variables (bio1, bio4, bio6, bio12, bio14 and bio15) and topographic heterogeneity (TOPO) as covariates. Species assemblages included in this analysis were under matched climate conditions between the two continents (i.e. grid cells located in the inner box of Fig. 2B).

	Tropical and extra-tropical				Tropical only			
Source	SS	df	*F*	*P*	SS	df	*F*	*P*
Standardized phylogenetic diversity (AF < SA)								
Continent	19.7	1	30.2	<0.001	9.7	1	19.6	<0.001
bio1	2.1	1	3.2	0.076	<0.1	1	<0.1	0.943
bio4	10.8	1	16.5	<0.001	<0.1	1	<0.1	0.974
bio6	<0.1	1	0.0	0.919	1.2	1	2.3	0.128
bio12	18.0	1	27.6	<0.001	5.8	1	11.7	0.001
bio14	0.1	1	0.2	0.654	<0.1	1	<0.1	0.866
bio15	1.0	1	1.5	0.227	0.2	1	0.4	0.531
TOPO	24.0	1	36.8	<0.001	5.8	1	11.6	0.001
Error	183.1	281			67.4	136		
Standardized mean phylogenetic distance (AF < SA)								
Continent	35.5	1	5.8	0.016	24.9	1	5.3	0.023
bio1	125.1	1	20.6	<0.001	1.1	1	0.2	0.622
bio4	60.9	1	10.0	0.002	6.2	1	1.3	0.252
bio6	65.2	1	10.7	0.001	2.1	1	0.5	0.502
bio12	189.6	1	31.3	<0.001	79.6	1	16.9	<0.001
bio14	1.5	1	0.2	0.623	4.2	1	0.9	0.346
bio15	26.5	1	4.4	0.038	0.9	1	0.2	0.669
TOPO	88.8	1	14.6	<0.001	6.8	1	1.5	0.230
Error	1705.0	281			639.8	136		

Note that data of phylogenetic diversity used in the analyses were in the unit of 1000 million years, and data of mean phylogenetic distance used in the analyses were in the unit of million years. Climatic variables bio1, bio12, bio6, bio14, bio4, and bio15 represent, respectively, mean annual temperature, annual precipitation, minimum temperature of the coldest month, precipitation during the driest month, temperature seasonality, and precipitation seasonality.

Table S6. Analysis of covariance of standardized phylogenetic diversity and standardized mean phylogenetic distance (based on 500 angiosperm species randomly selected from each botanical country) in Africa (AF) and South America (SA) with continent (AF versus SA) as the main effect and climate variables (bio1, bio4, bio6, bio12, bio14 and bio15) and topographic heterogeneity (TOPO) as covariates. Species assemblages included in this analysis were under matched climate conditions between the two continents (i.e. botanical countries located in the inner box of Fig. 2A).

	Tropical and extra-tropical				Tropical only			
Source	SS	df	*F*	*P*	SS	df	*F*	*P*
Standardized phylogenetic diversity (AF < SA)								
Continent	2.1	1	6.6	0.013	1.7	1	8.3	0.008
bio1	9.6	1	29.3	<0.001	1.9	1	9.2	0.005
bio4	7.9	1	24.3	<0.001	1.4	1	6.7	0.016
bio6	4.5	1	13.9	<0.001	0.9	1	4.5	0.045
bio12	8.2	1	25.3	<0.001	3.2	1	15.5	0.001
bio14	0.1	1	0.2	0.632	0.1	1	0.3	0.594
bio15	0.5	1	1.6	0.215	0.2	1	1.0	0.336
TOPO	1.4	1	4.3	0.044	0.9	1	4.4	0.047
Error	16.7	51			5.2	25		
Standardized mean phylogenetic distance (AF < SA)								
Continent	30.7	1	11.0	0.002	42.0	1	19.7	<0.001
bio1	50.2	1	17.9	<0.001	2.2	1	1.0	0.320
bio4	25.4	1	9.1	0.004	4.7	1	2.2	0.149
bio6	25.6	1	9.1	0.004	0.5	1	0.3	0.618
bio12	30.8	1	11.0	0.002	9.1	1	4.3	0.049
bio14	0.4	1	0.1	0.718	0.4	1	0.2	0.651
bio15	11.7	1	4.2	0.046	0.1	1	0.1	0.815
TOPO	0.8	1	0.3	0.594	2.5	1	1.2	0.287
Error	142.8	51			53.4	25		

Note that data of phylogenetic diversity used in the analyses were in the unit of 1000 million years, and data of mean phylogenetic distance used in the analyses were in the unit of million years. Climatic variables bio1, bio12, bio6, bio14, bio4, and bio15 represent, respectively, mean annual temperature, annual precipitation, minimum temperature of the coldest month, precipitation during the driest month, temperature seasonality, and precipitation seasonality.

Table S7. The first two principal components (PC) of a principal component analysis based on the correlation matrix between climate variables for regional floras (N = 422) in Africa and South America. Values in the lower part of the table are the eigenvectors of the two principal components.

	PC1	PC2
Eigenvalue	3.127	1.923
Percentage of variance	52.123	32.051
Cumulative % of variance	52.123	84.174
Eigenvectors		
bio1	0.297	0.546
bio12	0.493	-0.223
bio6	0.473	0.378
bio14	0.375	-0.454
bio4	-0.471	-0.188
bio15	-0.288	0.518

Climatic variables bio1, bio12, bio6, bio14, bio4, and bio15 represent, respectively, mean annual temperature, annual precipitation, minimum temperature of the coldest month, precipitation during the driest month, temperature seasonality, and precipitation seasonality.

REFERENCES

1. P. H. Raven, R. E. Gereau, P. B. Phillipson, C. Chatelain, C. N. Jenkins, C. Ulloa Ulloa, The distribution of biodiversity richness in the tropics *Sci. Adv.* **6**, eabc6228 (2020).

2. P. H. Raven, D. I. Axelrod, History of the flora and fauna in Latin America. *Am. Sci.* **63**, 420–429 (1975).

3. M. R. Carvalho, C. Jaramillo, F. de la Parra, D. Caballero-Rodríguez, F. Herrera, S. Wing, B. L. Turner, C. D'Apolito, M. Romero-Báez, P. Narváez, C. Martínez, M. Gutierrez, C. Labandeira, G. Bayona, M. Rueda, M. Paez-Reyes, D. Cárdenas, Á. Duque, J. L. Crowley, C. Santos, D. Silvestro, Extinction at the end-Cretaceous and the origin of modern Neotropical rainforests. *Science* **372**, 63–68 (2021).

4. P. V. Fine, R. H. Ree, Evidence for a time-integrated species-area effect on the latitudinal gradient in tree diversity. *Am. Nat.* **168**, 796–804 (2006).

5. P. L. S. de Miranda, K. G. Dexter, M. D. Swaine, A. T. de Oliveira-Filho, O. J. Hardy, A. Fayolle, Dissecting the difference in tree species richness between Africa and South America. *Proc. Natl. Acad. Sci. U.S.A.* **119**, e2112336119 (2022).

6. H. Qian, Y. Zhou, J. Zhang, Y. Jin, T. Deng, S. Cheng, A synthesis of botanical informatics for vascular plants in Africa. *Eco. Inform.* **64**, 101382 (2021).

7. C. Ulloa Ulloa, P. Acevedo-Rodriguez, S. Beck, M. J. Belgrano, R. Bernal, P. E. Berry, L. Brako, M. Celis, G. Davidse, R. C. Forzza, S. R. Gradstein, O. Hokche, B. Leon, S. Leon-Yanez, R. E. Magill, D. A. Neill, M. Nee, P. H. Raven, H. Stimmel, M. T. Strong, J. L. Villasenor, J. L. Zarucchi, F. O. Zuloaga, P. M. Jorgensen, An integrated assessment of the vascular plant species of the Americas. *Science* **358**, 1614–1617 (2017).

8. J. W. Slik, V. Arroyo-Rodriguez, S. Aiba, P. Alvarez-Loayza, L. F. Alves, P. Ashton, P. Balvanera, M. L. Bastian, P. J. Bellingham, E. van den Berg, L. Bernacci, P. da Conceicao Bispo, L. Blanc, K. Bohning-Gaese, P. Boeckx, F. Bongers, B. Boyle, M. Bradford, F. Q. Brearley, M. Breuer-Ndoundou Hockemba, S. Bunyavejchewin, D. Calderado Leal Matos, M. Castillo-Santiago, E. L.

Catharino, S. L. Chai, Y. Chen, R. K. Colwell, R. L. Chazdon, C. Clark, D. B. Clark, D. A. Clark, H. Culmsee, K. Damas, H. S. Dattaraja, G. Dauby, P. Davidar, S. J. DeWalt, J. L. Doucet, A. Duque, G. Durigan, K. A. Eichhorn, P. V. Eisenlohr, E. Eler, C. Ewango, N. Farwig, K. J. Feeley, L. Ferreira, R. Field, A. T. de Oliveira Filho, C. Fletcher, O. Forshed, G. Franco, G. Fredriksson, T. Gillespie, J. F. Gillet, G. Amarnath, D. M. Griffith, J. Grogan, N. Gunatilleke, D. Harris, R. Harrison, *A. Hector*, J. Homeier, N. Imai, A. Itoh, P. A. Jansen, C. A. Joly, B. H. de Jong, K. Kartawinata, E. Kearsley, D. L. Kelly, D. Kenfack, M. Kessler, K. Kitayama, R. Kooyman, E. Larney, Y. Laumonier, S. Laurance, W. F. Laurance, M. J. Lawes, I. L. Amaral, S. G. Letcher, J. Lindsell, X. Lu, A. Mansor, A. Marjokorpi, E. H. Martin, H. Meilby, F. P. Melo, D. J. Metcalfe, V. P. Medjibe, J. P. Metzger, J. Millet, D. Mohandass, J. C. Montero, M. de Morisson Valeriano, B. Mugerwa, H. Nagamasu, R. Nilus, S. Ochoa-Gaona, Onrizal, N. Page, P. Parolin, M. Parren, N. Parthasarathy, E. Paudel, A. Permana, M. T. Piedade, N. C. Pitman, L. Poorter, A. D. Poulsen, J. Poulsen, J. Powers, R. C. Prasad, J. P. Puyravaud, J. C. Razafimahaimodison, J. Reitsma, J. R. Dos Santos, W. Roberto Spironello, H. Romero-Saltos, F. Rovero, A. H. Rozak, K. Ruokolainen, E. Rutishauser, F. Saiter, P. Saner, B. A. Santos, F. Santos, S. K. Sarker, M. Satdichanh, C. B. Schmitt, J. Schongart, M. Schulze, M. S. Suganuma, D. Sheil, E. da Silva Pinheiro, P. Sist, T. Stevart, R. Sukumar, I. F. Sun, T. Sunderland, H. S. Suresh, E. Suzuki, M. Tabarelli, J. Tang, N. Targhetta, I. Theilade, D. W. Thomas, P. Tchouto, J. Hurtado, R. Valencia, J. L. van Valkenburg, T. Van Do, R. Vasquez, H. Verbeeck, V. Adekunle, S. A. Vieira, C. O. Webb, T. Whitfeld, S. A. Wich, J. Williams, F. Wittmann, H. Woll, X. Yang, C. Y. Adou Yao, S. L. Yap, T. Yoneda, R. A. Zahawi, R. Zakaria, R. Zang, R. L. de Assis, B. Garcia Luize, E. M. Venticinque, An estimate of the number of tropical tree species. *Proc. Natl Acad. Sci. U. S. A.* **112**, 7472–7477 (2015).

9. P. W. Richards, "Africa, the odd man out?" in *Tropical Forest Ecosystems of Africa and South America: A Comparative Review*, B. J. Meggers, E. S. Ayensu, W. D. Duckworth, Eds. (Smithsonian Institution Press, 1973), pp. 21–26.

10. T. L. P. Couvreur, Odd man out: Why are there fewer plant species in African rain forests? *Plant Syst. Evol.* **301**, 1299–1313 (2015).

11. O. Hagen, A. Skeels, R. E. Onstein, W. Jetz, L. Pellissier, Earth history events shaped the evolution of uneven biodiversity across tropical moist forests. *Proc. Natl. Acad. Sci. U.S.A.* **118**, e2026347118 (2021).

12. R. Cazzolla Gatti, P. B. Reich, J. G. P. Gamarra, T. Crowther, C. Hui, A. Morera, J. F. Bastin, S. de-Miguel, G. J. Nabuurs, J. C. Svenning, J. M. Serra-Diaz, C. Merow, B. Enquist, M. Kamenetsky, J. Lee, J. Zhu, J. Fang, D. F. Jacobs, B. Pijanowski, A. Banerjee, R. A. Giaquinto, G. Alberti, A. M. Almeyda Zambrano, E. Alvarez-Davila, A. Araujo-Murakami, V. Avitabile, G. A. Aymard, R. Balazy, C. Baraloto, J. G. Barroso, M. L. Bastian, P. Birnbaum, R. Bitariho, J. Bogaert, F. Bongers, O. Bouriaud, P. H. S. Brancalion, F. Q. Brearley, E. N. Broadbent, F. Bussotti, W. Castro da Silva, R. G. Cesar, G. Cesljar, *V. Chama* Moscoso, H. Y. H. Chen, E. Cienciala, C. J. Clark, D. A. Coomes, S. Dayanandan, M. Decuyper, L. E. Dee, J. Del Aguila Pasquel, G. Derroire, M. N. K. Djuikouo, T. Van Do, J. Dolezal, I. D. Dordevic, J. Engel, T. M. Fayle, T. R. Feldpausch, J. K. Fridman, D. J. Harris, A. Hemp, G. Hengeveld, B. Herault, M. Herold, T. Ibanez, A. M. Jagodzinski, B. Jaroszewicz, K. J. Jeffery, V. K. Johannsen, T. Jucker, A. Kangur, V. N. Karminov, K. Kartawinata, D. K. Kennard, S. Kepfer-Rojas, G. Keppel, M. L. Khan, P. K. Khare, T. J. Kileen, H. S. Kim, H. Korjus, A. Kumar, A. Kumar, D. Laarmann, N. Labriere, M. Lang, S. L. Lewis, N. Lukina, B. S. Maitner, Y. Malhi, A. R. Marshall, O. V. Martynenko, A. L. Monteagudo Mendoza, P. V. Ontikov, E. Ortiz-Malavasi, N. C. Pallqui Camacho, A. Paquette, M. Park, N. Parthasarathy, P. L. Peri, P. Petronelli, S. Pfautsch, O. L. Phillips, N. Picard, D. Piotto, L. Poorter, J. R. Poulsen, H. Pretzsch, H. Ramirez-Angulo, Z. Restrepo Correa, M. Rodeghiero, R. D. P. Rojas Gonzales, S. G. Rolim, F. Rovero, E. Rutishauser, P. Saikia, C. Salas-Eljatib, D. Schepaschenko, M. Scherer-Lorenzen, V. Seben, M. Silveira, F. Slik, B. Sonke, A. F. Souza, K. J. Sterenczak, M. Svoboda, H. Taedoumg, N. Tchebakova, J. Terborgh, E. Tikhonova, A. Torres-Lezama, F. van der Plas, R. Vasquez, H. Viana, A. C. Vibrans, E. Vilanova, V. A. Vos, H. F. Wang, B. Westerlund, L. J. T. White, S. K. Wiser, T. Zawila-Niedzwiecki, L. Zemagho, Z. X. Zhu, I. C. Zo-Bi, J. Liang, The number of tree species on Earth. *Proc. Natl. Acad. Sci. U. S. A.* **119**, e2115329119 (2022).

13. I. Parmentier, Y. Malhi, B. Senterre, R. J. Whittaker, A. Alonso, M. P. B. Balinga, A. Bakayoko, F. Bongers, C. Chatelain, J. A. Comiskey, R. Cortay, M.-N. D. Kamdem, J.-L. Doucet, L. Gautier, W. D. Hawthorne, Y. A. Issembe, F. N. Kouamé, L. A. Kouka, M. E. Leal, J. Lejoly, S. L. Lewis, L. Nusbaumer, M. P. E. Parren, K. S.-H. Peh, O. L. Phillips, L. Poorter, D. Sheil, B. Sonké, M. S. M.

Sosef, T. C. H. Sunderland, J. Stropp, H. ter Steege, M. D. Swaine, M. G. P. Tchouto, B. S. V. Gemerden, J. L. C. H. Van Valkenburg, H. Wöll, The odd man out? Might climate explain the lower tree α-diversity of African rain forests relative to Amazonian rain forests? *J. Ecol.* **95**, 1058-1071 (2007).

14. R. J. Morley, K. Richards, Gramineae cuticle: a key indicator of Late Cenozoic climatic change in the Niger Delta. *Rev.palaeobot. Palynol.* **77**, 119–127 (1993).

15. R. J. Morley, *Origin and Evolution of Tropical Rain Forests* (John Wiley and Sons 2000), pp. 362.

16. H. P. Linder, The evolution of African plant diversity. *Front. Ecol. Evol.* **2**, 38 (2014).

17. E. D. Currano, B. F. Jacobs, A. D. Pan, Is Africa really an "odd man out"? Evidence for diversity decline across the Oligocene-Miocene boundary. *IJPS* **182**, 551–563 (2021).

18. R. Wei, Q. Xiang, H. Schneider, M. A. Sundue, M. Kessler, P. W. Kamau, A. Hidayat, X. Zhang, Eurasian origin, boreotropical migration and transoceanic dispersal in the pantropical fern genusDiplazium(Athyriaceae). *J. Biogeogr.* **42**, 1809–1819 (2015).

19. A. Carta, L. Peruzzi, S. Ramírez-Barahona, A global phylogenetic regionalization of vascular plants reveals a deep split between Gondwanan and Laurasian biotas. *New Phytol.* **233**, 1494–1504 (2022).

20. H. Qian, Y. Jin, R. E. Ricklefs, Phylogenetic diversity anomaly in angiosperms between eastern Asia and eastern North America. *Proc. Natl. Acad. Sci. U.S.A.* **114**, 11452–11457 (2017).

21. D. P. Faith, Conservation evaluation and phylogenetic diversity. *Biol. Conserv.* **61**, 1–10 (1992).

22. T. J. Davies, L. B. Buckley, Phylogenetic diversity as a window into the evolutionary and biogeographic histories of present-day richness gradients for mammals. *Philos. Trans. R. Soc. Lond. B. Biol. Sci.* **366**, 2414–2425 (2011).

23. M. W. Cadotte, T. J. Davies, *Phylogenies in Ecology: A Guide to Concepts and Methods* (Princeton University Press, 2016).

24. M. Lavin, M. Thulin, J. N. Labat, R. T. Pennington, Africa, the odd man out: molecular biogeography of dalbergioid legumes (Fabaceae) suggests otherwise. *Syst. Bot.* **25**, 449–467 (2000).

25. L. Cai, Z. Xi, A. M. Amorim, M. Sugumaran, J. S. Rest, L. Liu, C. C. Davis, Widespread ancient whole genome duplications in Malpighiales coincide with Eocene global climatic upheaval. *New Phytol.* **221**, 565–576 (2019).

26. L. M. Cisneros, K. R. Burgio, L. M. Dreiss, B. T. Klingbeil, B. D. Patterson, S. J. Presley, M. R. Willig, Multiple dimensions of bat biodiversity along an extensive tropical elevational gradient. *J. Anim. Ecol.* **83**, 1124-1136 (2014).

27. L. M. Lu, L. F. Mao, T. Yang, J. F. Ye, B. Liu, H. L. Li, M. Sun, J. T. Miller, S. Mathews, H. H. Hu, Y. T. Niu, D. X. Peng, Y. H. Chen, S. A. Smith, M. Chen, K. L. Xiang, C. T. Le, V. C. Dang, A. M. Lu, P. S. Soltis, D. E. Soltis, J. H. Li, Z. D. Chen, Evolutionary history of the angiosperm flora of China. *Nature* **554**, 234–238 (2018).

28. B. D. Mishler, N. Knerr, C. E. Gonzalez-Orozco, A. H. Thornhill, S. W. Laffan, J. T. Miller, Phylogenetic measures of biodiversity and neo- and paleo-endemism in Australian Acacia. *Nat. Commun.* **5**, 4473 (2014).

29. M. B. De Souza Cortez, R. A. Folk, C. J. Grady, J. P. Spoelhof, S. A. Smith, D. E. Soltis, P. S. Soltis, Is the age of plant communities predicted by the age, stability and soil composition of the underlying landscapes? An investigation of OCBILs. *Biol. J. Linn. Soc.* **133**, 297-316 (2021).

30. L. M. J. Dagallier, S. B. Janssens, G. Dauby, A. Blach-Overgaard, B. A. Mackinder, V. Droissart, J. C. Svenning, M. S. M. Sosef, T. Stevart, D. J. Harris, B. Sonke, J. J. Wieringa, O. J. Hardy, T. L. P. Couvreur, Cradles and museums of generic plant diversity across tropical Africa. *New Phytol.* **225**, 2196–2213 (2020).

31. P. R. Kellar, D. L. Ahrendsen, S. K. Aust, A. R. Jones, J. C. Pires, Biodiversity comparison among phylogenetic diversity metrics and between three North American prairies. *Appl. Plant Sci.* **3**, 1400108 (2015).

32. L. Macheriotou, A. Rigaux, S. Derycke, A. Vanreusel, Phylogenetic clustering and rarity imply risk of local species extinction in prospective deep-sea mining areas of the Clarion-Clipperton Fracture Zone. *Proc. Biol.l Sci.* **287**, 20192666 (2020).

33. F. Villalobos, F. Carotenuto, P. Raia, J. A. F. Diniz-Filho, Phylogenetic fields through time: Temporal dynamics of geographical co-occurrence and phylogenetic structure within species ranges. *Philos.ophical Trans. R. Soc. Lond. B. Sci.* **371**, 20150220 (2016).

34. D. V. Pio, O. Broennimann, T. G. Barraclough, G. Reeves, A. G. Rebelo, W. Thuiller, A. Guisan, N. Salamin, Spatial predictions of phylogenetic diversity in conservation decision making. *Conserv. Biol.* **25**, 1229–1239 (2011).

35. H. Qian, T. Deng, Geographic patterns and climate correlates of the deviation between phylogenetic and taxonomic diversity for angiosperms in China. *Biol. Conserv.* **262**, 109291 (2021).

36. R. A. Scherson, A. A. Albornoz, A. S. Moreira-Munoz, R. Urbina-Casanova, Endemicity and evolutionary value: A study of Chilean endemic vascular plant genera. *Ecol. Evol.* **4**, 806 (2014), 816.

37. C. Jaramillo, "Evolution of the Isthmus of Panama: biological, paleoceanographic, and paleoclimatological implications" in *Mountains, Climate and Biodiversity,* C. Hoorn, A. Antonelli, Eds. (John Wiley & Sons, 2018), pp. 323–338.

38. M. O. Woodburne, The Great American Biotic Interchange: dispersals, tectonics, climate, sea level and holding pens. *J. Mamm. Evol.* **17**, 245–264 (2010).

39. C. Hughes, R. Eastwood, Island radiation on a continental scale: Exceptional rates of plant diversification after uplift of the Andes. *Proc. Natl. Acad. Sci. U. S. A.* **103**, 10334-10339 (2006).

40. P. Goldblatt, Floristic diversity in the Cape Flora of South Africa. *Biodivers. Conserv.* **6**, 359–377 (1997).

41. D. L. Rabosky, Likelihood methods for detecting temporal shifts in diversification rates. *Evolution* **60**, 1152–1164 (2006).

42. S. Louca, M. W. Pennell, Extant timetrees are consistent with a myriad of diversification histories. *Nature* **580**, 502–505 (2020).

43. A. J. Helmstetter, S. Glemin, J. Kafer, R. Zenil-Ferguson, H. Sauquet, H. de Boer, L. M. J. Dagallier, N. Mazet, E. L. Reboud, T. L. P. Couvreur, F. L. Condamine, Pulled diversification rates, lineages-through-time plots, and modern macroevolutionary modeling. *Syst. Biol.* **71**, 758–773 (2022).

44. A. H. Gentry, Neotropical floristic diversity: Phytogeographical connections between Central and South America, Pleistocene climatic fluctuations, or an accident of the Andean orogeny? *Ann. Mo. Bot. Gard.* **69**, 557–593 (1982).

45. C. D. Bacon, D. Silvestro, C. Jaramillo, B. T. Smith, P. Chakrabarty, A. Antonelli, Biological evidence supports an early and complex emergence of the Isthmus of Panama. *Proc. Natl. Acad. Sci. U.S.A.* **112**, 6110–6115 (2015).

46. S. Cody, J. E. Richardson, V. Rull, C. Ellis, R. T. Pennington, The great American biotic interchange revisited. *Ecography* **33**, 326–332 (2010).

47. A. Kremer, A. L. Hipp, Oaks: An evolutionary success story. *New Phytol.* **226**, 987–1011 (2020).

48. A. L. Hipp, P. S. Manos, M. Hahn, M. Avishai, C. Bodenes, J. Cavender-Bares, A. A. Crowl, M. Deng, T. Denk, S. Fitz-Gibbon, O. Gailing, M. S. Gonzalez-Elizondo, A. Gonzalez-Rodriguez, G. W. Grimm, X. L. Jiang, A. Kremer, I. Lesur, J. D. McVay, C. Plomion, H. Rodriguez-Correa, E. D. Schulze, M. C. Simeone, V. L. Sork, S. Valencia-Avalos, Genomic landscape of the global oak phylogeny. *New Phytol.* **226**, 1198–1212 (2020).

49. W. L. Crepet, K. C. Nixon, M. A. Gandolfo, Fossil evidence and phylogeny: The age of major angiosperm clades based on mesofossil and macrofossil evidence from Cretaceous deposits. *Am. J. Bot.* **91**, 1666–1682 (2004).

50. W. Küper, J. H. Sommer, J. C. Lovett, J. Mutke, H. P. Linder, H. J. Beentje, R. Renaat Sylva Angèle Rosine Van, C. Chatelain, M. Sosef, W. Barthlott, Africa's hotspots of biodiversity redefined. *Ann. Mo. Bot. Gard.* **91**, 525–535 (2004).

51. H. P. Linder, The radiation of the Cape flora, southern Africa. *Biol. Rev.* **78**, 597–638 (2003).

52. V. L. Rivera, J. L. Panero, E. E. Schilling, B. S. Crozier, M. D. Moraes, Origins and recent radiation of Brazilian Eupatorieae (Asteraceae) in the eastern Cerrado and Atlantic Forest. *Mol. Phylogenet. Evol.* **97**, 90–100 (2016).

53. T. M. Machado, O. Loiseau, M. Paris, A. Weigand, L. M. Versieux, J. R. Stehmann, C. Lexer, N. Salamin, Systematics of *Vriesea* (Bromeliaceae): Phylogenetic relationships based on nuclear gene and partial plastome sequences. *Bot. J. Linn. Soc.* **192**, 656–674 (2020).

54. M. Perret, A. Chautems, A. O. De Araujo, N. Salamin, Temporal and spatial origin of Gesneriaceae in the New World inferred from plastid DNA sequences. *Bot. J. Linn. Soc.* **171**, 61–79 (2013).

55. R. K. Brummitt, *World Geographical Scheme for Recording Plant Distributions* (Hunt Institute for Botanical Documentation, Carnegie Mellon University, ed. 2, 2001).

56. B. H. Daru, T. J. Davies, C. G. Willis, E. K. Meineke, A. Ronk, M. Zobel, M. Partel, A. Antonelli, C. C. Davis, Widespread homogenization of plant communities in the Anthropocene. *Nat. Commun.* **12**, 6983 (2021).

57. M. Tietje, A. Antonelli, W. J. Baker, R. Govaerts, S. A. Smith, W. L. Eiserhardt, Global variation in diversification rate and species richness are unlinked in plants. *Proc. Natl. Acad. Sci. U.S.A.* **119**, e2120662119 (2022).

58. R. Govaerts, E. Nic Lughadha, N. Black, R. Turner, A. Paton, The World Checklist of Vascular Plants, a continuously updated resource for exploring global plant diversity. *Sci. Data* **8**, 215 (2021).

59. M. J. M. Brown, B. E. Walker, N. Black, R. H. A. Govaerts, I. Ondo, R. Turner, E. rWCVP: A companion R package for the World Checklist of Vascular Plants. *New Phytol.*, 37289204 (2023).

60. J. Zhang, H. Qian, U.Taxonstand: U.Taxonstand: An R package for standardizing scientific names of plants and animals. *Plant Divers.* **45**, 1–5 (2023).

61. Y. Jin, H. Qian, V.V.PhyloMaker2: An updated and enlarged R package that can generate very large phylogenies for vascular plants. *Plant Divers.* **44**, 335–339 (2022).

62. Y. Jin, H. Qian, U.PhyloMaker: U.PhyloMaker: An R package that can generate large phylogenetic trees for plants and animals. *Plant Divers.* **45**, 347–352 (2023).

63. Y. Jin, H. Qian, V.PhyloMaker: An R package that can generate very large phylogenies for vascular plants. *Ecography* **42**, 1353–1359 (2019).

64. S. A. Smith, J. W. Brown, Constructing a broadly inclusive seed plant phylogeny. *Am. J. Bot.* **105**, 302–314 (2018).

65. H. Qian, T. Deng, Species invasion and phylogenetic relatedness of vascular plants on the Qinghai-Tibet Plateau, the roof of the world. *Plant Divers.*, (2023).

66. H. Qian, J. Zhang, M. Jiang, Global patterns of taxonomic and phylogenetic diversity of flowering plants: Biodiversity hotspots and coldspots. *Plant Divers.* **45**, 265–271 (2023).

67. Y. Zhang, L. Qian, D. Spalink, L. Sun, J. Chen, H. Sun, Spatial phylogenetics of two topographic extremes of the Hengduan Mountains in southwestern China and its implications for biodiversity conservation. *Plant Divers.* **43**, 181–191 (2021).

68. Y.-D. Zhou, B. H. Boru, S.-W. Wang, Q.-F. Wang, Species richness and phylogenetic diversity of different growth forms of angiosperms across a biodiversity hotspot in the horn of Africa. *J. Syst. Evol.* **59**, 141–150 (2021).

69. H. Qian, Y. Jin, Are phylogenies resolved at the genus level appropriate for studies on phylogenetic structure of species assemblages? *Plant Divers.* **43**, 255–263 (2021).

70. H. Qian, R. Field, J. Zhang, J. Zhang, S. Chen, Phylogenetic structure and ecological and evolutionary determinants of species richness for angiosperm trees in forest communities in China. *J. Biogeogr.* **43**, 603–615 (2016).

71. J. W. F. Slik, J. Franklin, V. Arroyo-Rodriguez, R. Field, S. Aguilar, N. Aguirre, J. Ahumada, S. I. Aiba, L. F. Alves, A. K, A. Avella, F. Mora, C. G. Aymard, S. Baez, P. Balvanera, M. L. Bastian, J. F. Bastin, P. J. Bellingham, E. van den Berg, P. da Conceicao Bispo, P. Boeckx, K. Boehning-Gaese, F. Bongers, B. Boyle, F. Brambach, F. Q. Brearley, S. Brown, S. L. Chai, R. L. Chazdon, S. Chen, P. Chhang, G. Chuyong, C. Ewango, I. M. Coronado, J. Cristobal-Azkarate, H. Culmsee, K. Damas, H. S. Dattaraja, P. Davidar, S. J. DeWalt, H. Din, D. R. Drake, A. Duque, G. Durigan, K. Eichhorn, E. S. Eler, T. Enoki, A. Ensslin, A. B. Fandohan, N. Farwig, K. J. Feeley, M. Fischer, O. Forshed, Q. S. Garcia, S. C. Garkoti, T. W. Gillespie, J. F. Gillet, C. Gonmadje, I. Granzow-de la Cerda, D. M. Griffith, J. Grogan, K. R. Hakeem, D. J. Harris, R. D. Harrison, *A. Hector*, A. Hemp, J. Homeier, M. S. Hussain, G. Ibarra-Manriquez, I. F. Hanum, N. Imai, P. A. Jansen, C. A. Joly, S. Joseph, K. Kartawinata, E. Kearsley, D. L. Kelly, M. Kessler, T. J. Killeen, R. M. Kooyman, Y. Laumonier, S. G. Laurance, W. F. Laurance, M. J. Lawes, S. G. Letcher, J. Lindsell, J. Lovett, J. Lozada, X. Lu, A. M. Lykke, K. B. Mahmud, N. P. D. Mahayani, A. Mansor, A. R. Marshall, E. H. Martin, D. Calderado Leal Matos, J. A. Meave, F. P. L. Melo, Z. H. A. Mendoza, F. Metali, V. P. Medjibe, J. P. Metzger, T. Metzker, D. Mohandass, M. A. Munguia-Rosas, R. Munoz, E. Nurtjahy, E. L. de Oliveira, Onrizal, P. Parolin, M. Parren, N. Parthasarathy, E. Paudel, R. Perez, E. A. Perez-Garcia, U. Pommer, L. Poorter, L. Qie, M. T. F. Piedade, J. R. R. Pinto, A. D. Poulsen, J. R. Poulsen, J. S. Powers, R. C. Prasad, J. P. Puyravaud, O. Rangel, J. Reitsma, D. S. B. Rocha, S. Rolim, F. Rovero, A. Rozak, K. Ruokolainen, E. Rutishauser, G. Rutten, M. N. Mohd Said, F. Z. Saiter, P. Saner, B. Santos, J. R. Dos Santos, S. K. Sarker, C. B. Schmitt, J. Schoengart, M. Schulze, D. Sheil, P. Sist, A. F. Souza, W. R. Spironello, T. Sposito, R. Steinmetz, T. Stevart, M. S. Suganuma, R. Sukri, A. Sultana, R. Sukumar, T. Sunderland, Supriyadi, H. S. Suresh, E. Suzuki, M. Tabarelli, J. Tang, E. V. J. Tanner, N. Targhetta, I. Theilade, D. Thomas, J. Timberlake, M. de Morisson Valeriano, J. van Valkenburg, T. Van Do, H. Van Sam, J. H. Vandermeer, H. Verbeeck, O. R. Vetaas, V. Adekunle, S. A. Vieira, C. O. Webb, E. L. Webb, T. Whitfeld, S. Wich, J. Williams, S. Wiser, F. Wittmann, X. Yang, C. Y. Adou Yao, S. L. Yap, R. A. Zahawi, R. Zakaria, R. Zang, Phylogenetic classification of the world's tropical forests. *Proc. Natl. Acad. Sci. U.S.A* **115**, 1837–1842 (2018).

72. J. W. F. Slik, N. Raes, S.-I. Aiba, F. Q. Brearley, C. H. Cannon, E. Meijaard, H. Nagamasu, R. Nilus, G. Paoli, A. D. Poulsen, D. Sheil, E. Suzuki, J. L. C. H. v. Valkenburg, C. O. Webb, P.

Wilkie, S. Wulffraat, Environmental correlates for tropical tree diversity and distribution patterns in Borneo. *Divers. Distrib.* **15**, 523–532 (2009).

73. G. Tang, M. G. Zhang, C. Liu, Z. Zhou, W. Chen, J. W. F. Slik, Phylogenetic support for the Tropical Niche Conservatism Hypothesis despite the absence of a clear latitudinal species richness gradient in Yunnan's woody flora. *Biogeosci.* **11**, 7055–7077 (2014).

74. C. Tsirogiannis, B. Sandel, PhyloMeasures: a package for computing phylogenetic biodiversity measures and their statistical moments. *Ecography* **39**, 709–714 (2016).

75. S. A. Fritz, C. Rahbek, Global patterns of amphibian phylogenetic diversity. *J. Biogeogr.* **39**, 1373–1382 (2012).

76. F. Leprieur, C. Albouy, J. D. Bortoli, P. F. Cowman, D. R. Bellwood, D. Mouillot, Quantifying phylogenetic beta diversity: Distinguishing between ‘true’ turnover of lineages and phylogenetic diversity gradients. *PLOS ONE* **7**, e42760 (2012).

77. H. Qian, S. Qian, Geographic patterns of taxonomic and phylogenetic β-diversity of angiosperm genera in regional floras across the world. *Plant Divers.*, (2023).

78. R. Kooyman, M. Rossetto, C. Allen, W. Cornwell, Australian tropical and subtropical rain forest community assembly: Phylogeny, functional biogeography, and environmental gradients. *Biotropica* **44**, 668–679 (2012).

79. H. Qian, M. Kessler, T. Deng, Y. Jin, Patterns and drivers of phylogenetic structure of pteridophytes in China. *Glob. Ecol. Biogeogr.* **30**, 1835-1846 (2021).

80. P. Weigelt, W. D. Kissling, Y. Kisel, S. A. Fritz, D. N. Karger, M. Kessler, S. Lehtonen, J. C. Svenning, H. Kreft, Global patterns and drivers of phylogenetic structure in island floras. *Sci. Rep.* **5**, 12213 (2015).

81. D. N. Karger, O. Conrad, J. Böhner, T. Kawohl, H. Kreft, R. W. Soria-Auza, N. E. Zimmermann, H. P. Linder, M. Kessler, Climatologies at high resolution for the earth’s land surface areas. *Sci. Data* **4**, 170122 (2017).

82. B. McCune, M. J. Mefford, *PC-ORD - Multivariate Analysis of Ecological Data (Version 4.0)* (MjM Software Design, 1999).

83. Z. Wang, J. Fang, Z. Tang, X. Lin, Patterns, determinants and models of woody plant diversity in China. *Proc. Biol. Sci.* **278**, 2122–2132 (2011).

84. R. Field, E. M. O'Brien, R. J. Whittaker, Global models for predicting woody plant richness from climate: Development and evaluation. *Ecology* **86**, 2263–2277 (2005).

85. R. E. Ricklefs, H. Qian, P. S. White, The region effect on mesoscale plant species richness between eastern Asia and eastern North America. *Ecography* **27**, 129-136 (2004).

86. Effects of regional vs. ecological factors on plant species richness: An intercontinental analysis. *Ecology* **88**, 1440–1453 (2007).

87. L. Wilkinson, M. Hill, J. P. Welna, G. K. Birkenbeuel, *SYSTAT for Windows: Statistics* (SYSTAT Inc., 1992).
